# Seasonal diversity dynamics of a boreal zooplankton community under climate impact

**DOI:** 10.1007/s00442-022-05165-0

**Published:** 2022-04-26

**Authors:** Edwige Bellier, Steinar Engen, Thomas Correll Jensen

**Affiliations:** 1 Department of Arctic and Marine Biology, UiT The Arctic University of Norway, 9037 Tromsø, Norway; 2 Centre for Biodiversity Dynamics, Department of Mathematical Science, Norwegian University for Science and Technology, 7491 Trondheim, Norway; 3 Norwegian Institute for Nature Research, Sognsveien 68, 0855 Oslo, Norway; 4 Department of Natural Resources Science, University of Rhode Island, Kingston, RI 02881 USA

**Keywords:** Freshwater, Lognormal distribution, Similarity, Return to equilibrium, Time-series

## Abstract

Seasonality and long-term environmental variability affect species diversity through their effects on the dynamics of species. To investigate such effects, we fitted a dynamic and heterogeneous species abundance model generating the lognormal species abundance distribution to an assemblage of freshwater zooplankton sampled five times a year (June–October) during the ice-free period over 28 years (1990–2017) in Lake Atnsjøen (Norway). By applying a multivariate stochastic community dynamics model for describing the fluctuations in abundances, we show that the community dynamics was driven by environmental variability in spring (i.e., June). In contrast, community-level ecological heterogeneity is highest in autumn. The autumn months (i.e., September and October) that rearranged the community are most likely crucial months to monitor long-term changes in community structure. Indeed, noises from early summer are filtered away, making it easier to track long-term changes. The community returned faster towards equilibrium when ecological heterogeneity was the highest (i.e., in September and October). This occurred because of stronger density-regulation in months with highest ecological heterogeneity. The community responded to the long-term warming of water temperature with decreasing species diversity and increasing abundance. Unevenness associated with variabilities in abundances might affect species interactions within the community. These can have consequences for the stability and functioning of the ecosystem.

**Supplementary Information:**

The online version contains supplementary material available at 10.1007/s00442-022-05165-0.

## Introduction

The dynamics of biological diversity is governed by the direct effects of environmental variations on population growth and species interactions (Chase et al. 2002; Chesson 2000). A better understanding of the factors affecting the species’ coexistence within dynamics communities is essential to apprehend the complex consequences of global change on the ecosystem's functioning over short and long-term temporal scales. Patterns in species composition are influenced by four processes, such as selection, drift, speciation and dispersal (Vellend 2010). Species are added to the community by speciation and dispersal. Then, the relative abundances of the species are shaped by drift and selection and ongoing dispersal to drive community dynamics. The neutral theory (Hubbell 2001) intends to explain how species can coexist in a given ecosystem by assuming that interacting species are ecologically equivalent. Their relative abundances can only fluctuate because of the probabilistic nature of individual birth and death called demographic stochasticity and dispersal limitation. This theory is widely used because of its simplicity and provides assumptions and predictions on how species evolutionary adaptations (i.e., niche-based hypotheses) can be analyzed. A more general model of community dynamics has been proposed by Engen and Lande (1996) and Lande et al. (2003), where species are modeled as a set of correlated diffusion processes, with different growth rates, thus taking into account the basic ecology of the different species and their responses to environmental fluctuations. By linking the mathematical model and the statistical species abundance distribution model, spatial and temporal variation in the community correlation can be analyzed (Sæther et al. 2013). Furthermore, the variance of the log-normal species abundance distribution can be partitioned into components expressing the dynamics of the community, such as environmental stochasticity and ecological heterogeneity (Engen et al. 2002). This model was used to analyze a community of tropical butterflies (Engen et al. 2002; Lande et al. 2003), aquatic insects (Engen et al. 2011b), and freshwater zooplankton (Bellier et al. 2014). These studies showed that the relative abundances fluctuations were attributable to both common and species-specific responses to environmental fluctuations (environmental stochasticity) and differences in the basic ecology of each species (ecological heterogeneity among species). The ecological heterogeneity arises from differences among species in their density-independent growth rates *r* that produce differences in their equilibrium abundances. The population growth, reproduction and demographic characteristics are mediated by phenotypic characteristics, such as traits (Abrams 1995; Werner and Peacor 2003), determined by the fitness of an organism in given biotic and abiotic conditions (Litchman and Klausmeier 2008). Trait is considered as any measurable characteristics of an individual, including phenotype and demographic parameters, such as fecundity, growth or survival (McGill et al. 2006). Heterogeneity in species traits may act to cause variation among species in mean abundances across time e.g., ranging from relatively common to relatively rare. The ecological heterogeneity might play a crucial role in enhancing the resilience of an ecosystem’s trophic structure and stability during periods of major environmental change, such as global warming (Lande et al. 2003). Indeed, diversity may either lower (May 1973) or increase the population stability (Loreau 2010; McCann et al. 1998; Naeem and Li 1997) conditionally on the strength of the different interactions between species of the community. Besides, fluctuations of relative abundances within a community—the community dynamics—can also be influenced by seasonal shifts, leading to community structures fluctuating with the seasons. If some species are abundant at different times, changes in their responses to seasonality, such as changes in their phenology (e.g., the timing of hatching, arising from dormancy) might have consequences for community responses to climate change. Understanding this mechanism is vital to improve the forecast of species diversity changes in a variable environment (Forrest and Miller-Rushing 2010; Parmesan and Yohe 2003; Visser et al. 2010).

Investigating how community dynamics influence species diversity to reinforce the stability of an ecosystem requires long-term studies that include both species diversity and environmental changes (Magurran et al. 2010). However, data collections on whole communities and spanning over more than a couple of decades are rare in ecology. Yet, only a few studies analyze how species diversity dynamics change through the seasons and is affected by long-term environmental fluctuations. Such studies are essential to understand biodiversity changes and mitigate the effects of climate change (Tilman et al. 2006). A 10-year study of Ecuadorian fruit-feeding butterflies showed that the species diversity followed the seasonal rhythm of dry and wet seasons (Grøtan et al. 2012). In another study, Grøtan et al. (2014) showed that a community of butterflies from Costa-Rica had biannual cycles in species diversity. Still, community similarity had an annual cycle peaking in the driest months, and similarity did not decline with increasing time lag. Vasseur et al. (2005) demonstrated that the dynamics of the species of a phytoplankton community changed from synchronous to asynchronous fluctuations. These changes in species dynamics’ characteristics were driven by seasonal alternation in the factors limiting phytoplankton growth. Seasonal and long-term environmental variability can influence the species’ dynamics, which can affect the structure in abundances of a community over time (Guo et al. 2002).

In an environment with strong seasonality, species can cope with the seasonal environment, which leads to seasonal succession of species (Hu and Tessier 1995; Kenitz et al. 2017; Sommer et al. 1986; Yoshida et al. 2001). This ecological process (i.e., the seasonal succession of species) can generate community structure differences from one season to another. Investigating patterns of community structure across seasons requires long-term studies that include both species diversity and environmental changes. Zooplankton plays a key role in aquatic food webs, e.g., in the trophic transfer in food webs. Therefore, it is essential to understand how environmental variability affects the links between physical processes and zooplankton dynamics (Winder and Schindler 2004).

Moreover, the zooplankton communities respond quickly to changes in environmental conditions (Shurin et al. 2010). Especially, the phenology of zooplankton species (i.e., the seasonal succession of species) is determined by changes in temperature, light, food availability, thermal stratification, resource competition, predator–prey dynamics, and life-history traits (Sommer et al. 2012). In this study, we used a 28-year long-time series (1990–2017) of a zooplankton community collected monthly during the growing season (i.e., from June to October) in Lake Atnsjøen (Norway). The structure of communities is most often analyzed from characteristics of single samples at given localities without modeling the temporal or spatial differences. Single samples analyses model the shape of the distribution of abundance among species (Fisher et al. 1943; Matthews and Whittaker 2015; Preston 1948) but ignore that the underlying dynamics of species drive the composition of communities. Factors affecting the structure of communities may be more clearly identified using model-based assumptions about the underlying species-specific dynamics (Caswell 1976; Engen and Lande 1996; Etienne et al. 2007; Henderson and Magurran 2010; Hubbell 2001; Mutshinda et al. 2009). Non-neutral models allow for variation among species in one or more parameters influencing their dynamics as well as effects of a variable environment.

Therefore, in this study, we apply a stochastic community dynamics model that generates the lognormal species abundance distribution (Engen and Lande 1996). We then use this distribution to analyze the community dynamics and the seasons’ influence on the time it takes for the community to return to its equilibrium. The community is at its equilibrium when the species abundances fluctuate at a stable state. Our model defines the equilibrium as the inverse of the temporal autocorrelation of the species dynamics. The life cycle of the zooplankton is on the order of months. Several years of sampling allow an estimation of the strength of the intraspecific density dependence and the temporal autocorrelation of the community composition. By estimating the community’s autocorrelation function (see Eq. 2), we decompose the variance of the normal distribution of log abundances into three additive components (see Eq. 5). Expressing the log-variance of the species abundance distribution in function of the theoretical community dynamics model (Engen et al. 2011a; Engen and Lande 1996) allows deducing that the species-specific stochastic dynamics can explain the first component (see Eqs. 1–5). The ecological heterogeneity explains the second; and the third is explained by higher variability in species abundances than expected in a given time (i.e., overdispersion in sampling relative to the Poisson distribution) (see Eq. 5). We assume that species-specific niches influence community dynamics. Thus, we expect to find large contributions from the ecological heterogeneity and species-specific dynamics to the community dynamics. We hypothesize that species-specific contribution to community dynamics contradicts the assumption of an ecological equivalence among species made in the neutral theory. Analyzing this hypothesis enables us to better understand the processes that lead to fluctuations in the relative abundances of the species of a community. We examined the role of both seasonal fluctuations (i.e., fluctuations in spring (June), summer (July–August), and autumn (Sept–Oct)) and long-term environmental variations in the maintenance of species diversity through their influence on community dynamics. By analyzing the community dynamics over time independently at different seasons, we attempt to explore how seasonality influences community dynamics and species diversity fluctuations. We also aim to identify the environmental factors that might decrease long-term species diversity, resulting in consequences for the ecosystem’s functioning.

## Materials and methods

### Community dynamics model

The community dynamics model is based on a general stochastic theory, with colonization, speciation, extinction, density regulation and environmental stochasticity (Engen and Lande 1996). For data sets collected over a relatively short time period, the model can be approximated by a stationary model with a constant given number of species ignoring possible changes in species composition during the sampling period (Engen et al. 2011a). The model allows species abundances to change due to species-specific and common environmental stochasticity in the density-independent growth rate *r*. The abundances of species can also vary because of deterministic density dependence acting within species (Online Resource 1). These factors cause temporal fluctuations in relative abundances. This community dynamics model assumes that the Gompertz curve represents the density regulation within each species and that the environmental variances are constant, enabling to produce a lognormal species abundance distribution (Engen and Lande 1996). The link between the statistical model and the stochastic model is established through correlation in the noises generating the correlation in the bivariate lognormal model. We assume that the environmental noise is acting independently on each species. This allows reproductive rates and mortality of the species of the community to fluctuate independently over time. There is a noise term common to all species that is confounded with the mean abundances and have no effect on the distribution of relative abundances or correlations (Lande et al. 2003). Each species is described by a continuous diffusion process (i.e., an Ornstein–Uhlenbeck process) leading to the dynamics of log abundances *X*_*i*_ of species number *i* given by

1 $$\mathrm{d}{X}_{i}=\left({r}_{i}-\delta {X}_{i}\right)\mathrm{d}t+{\sigma }_{s}\mathrm{d}{B}_{i}\left(t\right)+{\sigma }_{c}\mathrm{d}{B}_{c}\left(t\right),$$

where *t* denotes time. Here *r*_*i*_ is the stochastic growth rate of species *i* in the absence of density regulation and *δ* is the strength of density-regulation, giving carrying capacity $K={e}^{{r}_{i}/\delta }$ varying among species. The function *B*_*i*_(*t*) and *B*_*c*_(*t*) denote random walks (i.e., Brownian motions) in time *t* with the properties *E*(d*B*(*t*)) = 0 and *E*(d*B*(*t*)^2^) = d*t*. The d*B*_*i*_(*t*) are noise component independent among species. The abundance of all species of the community are also affected in the same way by a noise d*B*_*c*_(*t*) which is common for all species. We assume a constant number of species during the period of data collection, thus, each *X*_*i*_ is normally distributed with mean value *r*_*i*_*/δ* and variance ${\sigma }_{e}^{2}/\left(2\delta \right).$ The log abundance (*X*_1_,* X*_2_, *…, X*_*n*_) is a sample from the normal distribution with mean *r*_*0*_*/δ* and variance ${\sigma }_{e}^{2}/\left(2\delta \right)+{\sigma }_{r}^{2}/{\delta }^{2}$, because *r*_*i*_ are themselves normally distributed in the community with mean *r*_0_, and variance ${\sigma }_{e}^{2}$ constitute the total environmental variance for each species. According to the community dynamics model (see Online Resource 1) and following Engen et al. (2011a), the correlation estimated between two samples from two communities with time *t* takes the form:

2 $${\rho }_{t}=\left({\rho }_{0}-{\rho }_{\infty }\right){e}^{-\delta t}+{\rho }_{\infty },$$

where

3 $${\rho }_{0}=\frac{{\sigma }_{s}^{2}/\left(2\delta \right)+{\sigma }_{r}^{2}/{\delta }^{2}}{{\sigma }_{s}^{2}/\left(2\delta \right)+{\sigma }_{r}^{2}/{\delta }^{2}+{\theta }^{2}},$$

and

4 $${\rho }_{\infty }=\frac{{\sigma }_{r}^{2}/\delta }{{\sigma }_{s}^{2}/\left(2\delta \right)+{\sigma }_{r}^{2}/\delta^{2} +{\theta }^{2}},$$

where ${\sigma }_{s}^{2}$ is the species specific noise which corresponds to stochastic components that are independent among species and *θ*^*2*^ quantifies the variability in species abundances which is higher than expected in a given time (i.e., the overdispersion component) (Online Resource 1). The total variance of the lognormal species abundance distribution ${\sigma }_{\mathrm{total}}^{2}={\sigma }_{s}^{2}/\left(2\delta \right)+{\sigma }_{r}^{2}/{\delta }^{2}+{\theta }^{2}$ can be estimated by fitting the univariate Poisson lognormal to each sample separately, because it is the variance parameter of this distribution. The ecological heterogeneity is expressed by ${\sigma }_{\mathrm{heter}}^{2}={\sigma }_{r}^{2}/{\delta }^{2}={\rho }_{\infty }{\sigma }_{\mathrm{total}}^{2},$ while ${\sigma }_{\mathrm{stoch}}^{2}={\sigma }_{s}^{2}/\left(2\delta \right)\left({\rho }_{0}-{\rho }_{\infty }\right){\sigma }_{\mathrm{total}}^{2}$ is generated by species specific environmental noise terms, where ${\sigma }_{s}^{2}$ are species specific noise terms (see Online Resource 1). The component due to overdispersion is ${\theta }^{2}=\left(1-{\rho }_{0}\right){\sigma }_{\mathrm{total}}^{2}$. By first estimating the autocorrelation function (Eq. 2), we partitioned the total variance of the lognormal distribution (i.e., ${\sigma }_{\mathrm{total}}^{2}$) into three additive components,

5 $${\sigma }_{\mathrm{total}}^{2}={\sigma }_{\mathrm{stoch}}^{2}+{\sigma }_{\mathrm{heter}}^{2}+{\theta }^{2}.$$

First, there is environmental component due to independent environmental noise terms with variance ${\sigma }_{s}^{2}$. The second term is the interspecies heterogeneity component due to the ecological heterogeneity determined by the variance ${\sigma }_{r}^{2}$ of the stochastic growth rates *r* of the different species *i* and $\delta$ the strength of density-regulation, while the third term reflects the sampling overdispersion. The return time to equilibrium $1/\delta$ can be considered as a quantitative measure of the stability in an ecosystem. Under the assumption of neutral dynamics ${\sigma }_{\mathrm{stoch}}^{2}={\sigma }_{\mathrm{heter}}^{2}=0$ and there will only be a small variance due to demographic noise.

### Species diversity and environmental fluctuations

Here, we use estimates of the total variance of the lognormal distribution ${\sigma }^{2}={\sigma }_{\mathrm{total}}^{2}$ as an inverse estimate of the evenness of the community (Bulmer 1974) denoted unevenness in this study. For a given number of species, *S*, the diversity is maximized when all species are equally abundant, and the diversity decreases with increasing unevenness in species abundances produced by increasing *σ*^*2*^ (Grøtan et al. 2012; Lande 1996). The evenness of plankton communities has been used to analyze the relationship between biodiversity and ecosystem functioning of aquatic systems (Filstrup et al. 2014; Hillebrand et al. 2008). We use *σ*^*2*^ as a species diversity measure, because it can be easily related to the community dynamics model (Engen et al. 2011a; Engen and Lande 1996). This is also demonstrated in section “Community dynamics model”, see Eqs. 1–5.

We apply a parametric approach assuming a lognormal distribution of actual species abundance and Poisson sampling within species (Fisher et al. 1943) (Online Resource 1). The distribution of the observed number of individuals among species then follows the Poisson lognormal distribution (Bulmer 1974), whereas joint samples from two communities follow the bivariate Poisson lognormal distribution (Engen et al. 2011b, 2002). The lognormal species abundance distribution often provides a good fit to data from species rich communities (Preston 1948) especially when sampling effects are included (Connolly et al. 2009; Engen et al. 2002; Grøtan et al. 2012; McGill et al. 2007; Preston 1948). The estimated correlation *ρ* of the natural log of actual species abundance distribution between two samples enabled to measure community similarity between each pair of years. The observed number of individuals for species *i* is ${N}_{i}\sim \mathrm{Poisson}\left({e}^{{X}_{i}}\right)$ and ${X}_{i}\sim N\left(\mu ,{\sigma }^{2}\right)$. The expected number of individuals *i* is then,

6 $$E\left({N}_{i}\right)=E\left(E\left({N}_{i}|X\right)\right)=E\left({e}^{X}\right)={e}^{\mu +\frac{1}{2}{\sigma }^{2}}.$$

We determined the relative importance of environmental variables on the variance *σ*^*2*^ by fitting generalized additive mixed model (GAMM) (Wood 2006). The Akaike Information Criterion (AIC) was used to evaluate each model’s fit and parsimony in the modelling process. The final model was obtained by eliminating variables with high *p* values, until the AIC became minimal.

### Similarity in time

When considering two communities jointly, either at two different locations or within the same community at two different timepoints, we assume that the log abundances in the pair of communities have the binormal distribution so that the lognormal model still fits each community separately (Engen et al. 2002). The correlation of this distribution then serves as a measurement of similarity between communities (Online Resource 2). Goodness-of-fit statistics of the observed species abundance distribution to the Poisson lognormal distribution can be obtained by comparing the likelihood when fitting the observed data with the bootstrap distribution of log likelihoods produced by simulating data and refitting the parameters (Grøtan et al. 2012). A lack of fit will be indicated if the log-likelihood of the data occurs towards one of the tails in the bootstrap distribution of log-likelihoods. The R-package poilog (Grøtan and Engen 2008) was used for estimating the parameters of the univariate and bivariate Poisson lognormal distribution. To reveal time-decay of similarity (Engen et al. 2011a), all pairwise estimates of the correlation parameter of the two-dimensional Poisson lognormal species abundance distribution were plotted against time lag. The schematic representation of the modelling approach to analyze the effect of environmental variability on species diversity through community dynamics is given in Fig. S1 in Online Resource 3.

### Study site and data collection

The oligotrophic Lake Atnsjøen is located in an Alpine–Boreal mountain area in south eastern Norway, 701 m a.s.l (Fig. S2 in Online Resource 4). The Lake is little affected by human activities and is covered by ice from late November to late May. The ice-cover length varied from 132 to 200 days over the study period (see Fig. S3 in Online Resource 3). The sampling took place during the ice-free period at 5 distinct periods (i.e., June, July, August, September and October) that were similar throughout the study from 1990 to 2017. Zooplankton samples were taken as two parallel plankton net hauls from 20 m to the surface. Sampling techniques and enumeration of the zooplankton are described by Halvorsen et al. (2004). For the enumeration, a subsample or successional subsamples were drawn from the original sample, where all species were identified and all individuals of each species were counted (Edmondson and Vinberg 1971). Then, the original sample was screened to determine the incidence of species not represented in the subsamples. The zooplankton community is composed of rotifers, copepods and cladocerans. The community is dominated by rotifers, such as *Polyartha vulgaris*, *Kellicottia longispina* and *Conochilus unicornis* which have short generation times (2–4 months). Among the crustaceans *Cyclops scutifer* and *Bosmina longispina* are the most abundant, other species such as *Holopedium gibberum* and *Daphnia longispina* are common (see Fig. S4 in Online Resource 4). In Lake Atnsjøen a larger part of the *Cyclops scutifer* population has a 1-year life cycle and a smaller part a 2-year cycle (Halvorsen et al. 2004). To analyze the variability of species diversity (i.e., unevenness, σ^2^) as a function of environmental fluctuations, we chose a set of five covariables (i.e., water temperature, water transparency, phytoplankton biomass, river run-off and duration of ice-cover). We selected these five covariates regarding their potential effects on species diversity (see Fig. S5 in Online Resource 4). We analyzed the effects of these covariates on the variability of species diversity using generalized additive mixed models (see Online Resource 5).

## Results

Our statistical analysis assumed a lognormal distribution of actual species abundance (Preston 1948) and Poisson sampling within species (Bulmer 1974; Engen et al. 2002; Lande et al. 2003). We tested if both replicated samples were issued of the same species abundance distribution overtime using a Kolmogorov–Smirnoff test (Sokal and Rohlf 1981). We hypothesized that the null hypothesis (*H*_0_) was that the frequency distributions of abundances of the two samples do not differ. The alternative hypothesis (*H*_a_) was that the frequency distributions of abundances of the two samples are different. The hypothesis (*H*_0_) was accepted (replicate A and replicate B, *D* = 0.078, *p* > 0.05). Thus, we hypothesized that the frequency distributions of abundances of the two samples do not differ*.* This allowed us the calculation of the correlation coefficients for a time lag of zero. We used one of the parallel samples (i.e., replicate A) to analyze the species distribution and community dynamics. The observed species abundance distribution for the pooled sample (i.e., with all the months over all the years of study) of about 7 million of individuals contained 32 species of copepods, cladocerans and rotifers. The expected proportion of species in abundance classes followed a Poisson-lognormal distribution (Fig. S6 in Online Resource 4). Parameter estimates for the lognormal species abundance distribution with their 95% confidence intervals obtained from a parametric bootstrap were $\widehat{\mu }$=8.152[6.297–9.606] and ${\widehat{\sigma }}^{2}$ = 15.376 [7.145–26.977]. The estimate of the approximate fraction of species revealed by the pooled sample was 0.983 and 32 species were observed, the estimate of the actual number of species in the community was $\widehat{S}$ ≈ 33.

### Seasonal fluctuations of species diversity

**Fig. 1:**
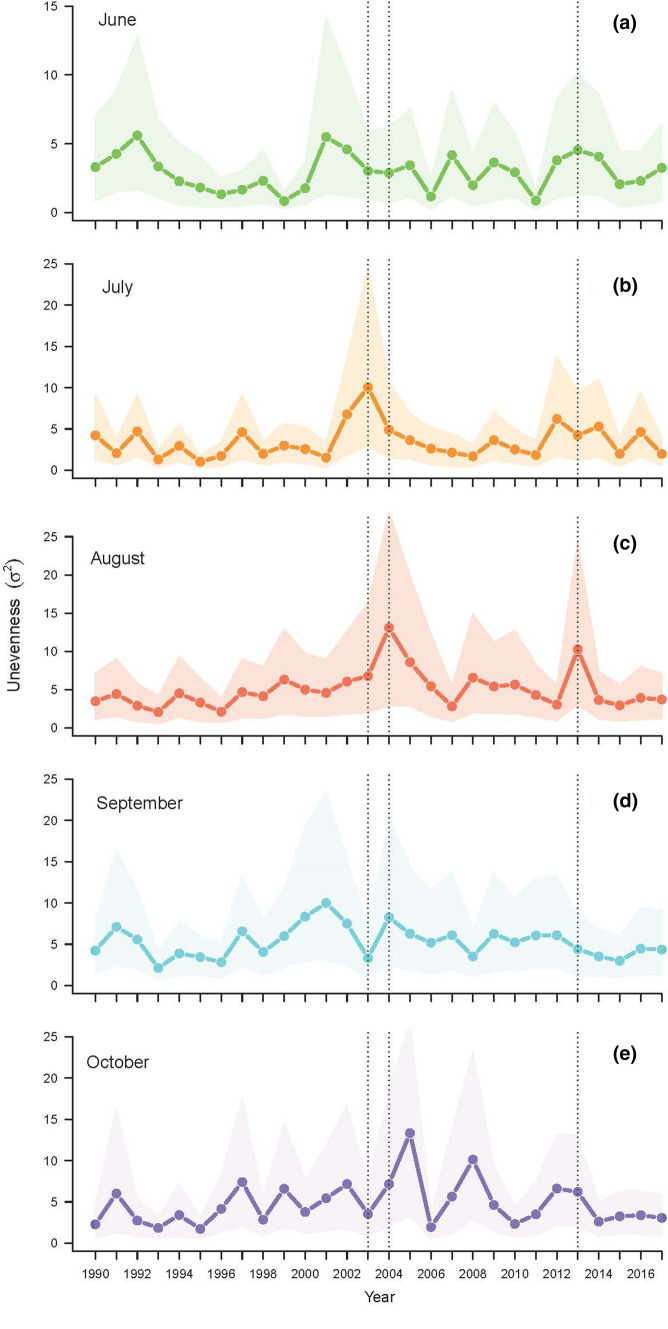
Unevenness estimates ${\widehat{\sigma }}^{2}$ as a function of years in **a** June, **b** July, **c** August, **d** September, and **e** October. The dotted lines highlight the years with a peak of unevenness during summer (i.e., June 2003, August 2004 and August 2013). The shaded areas correspond to the 95% confidence intervals

Species unevenness increased (i.e., *σ*^2^ increased) from June to October (Fig. 1a–e). The unevenness was higher in August, September and October than in June and July. A peak of unevenness (i.e., high *σ*^2^) was observed in July 2003 (Fig. 1b). This peak can be associated with a bloom in phytoplankton observed in July 2003 (Fig. S5f in Online Resource 4). The following months the diversity returned to a higher level (i.e., lower *σ*^2^). Peaks of unevenness (Fig. 1c–e) associated with phytoplankton blooms, were also observed in August 2004 and 2013. However, the diversity remained rather low the following months (Fig. 1d–e).

**Fig. 2:**
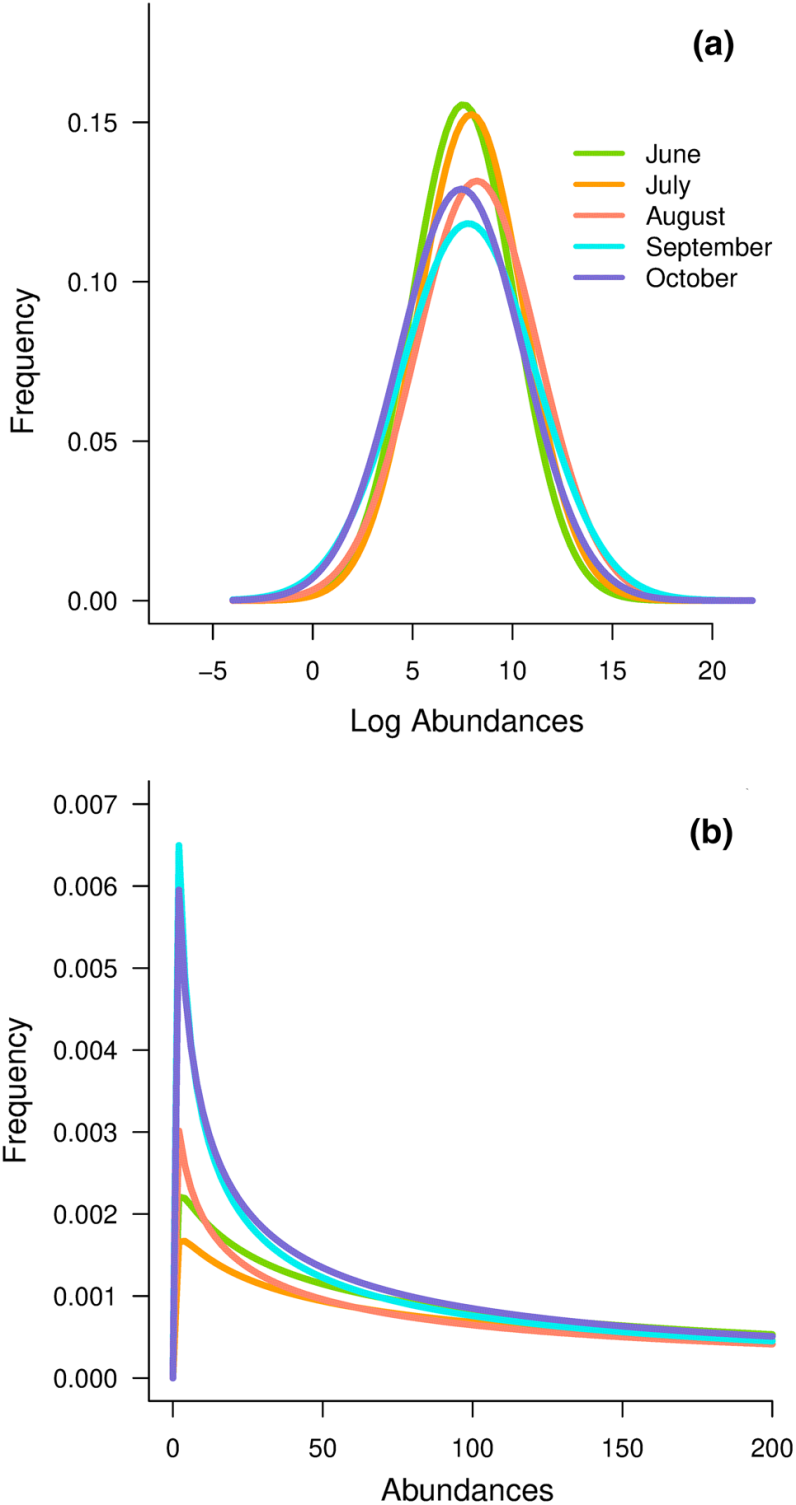
**a** Normal density of the species log abundances distribution estimated from $\widehat{\mu }$ and ${\widehat{\sigma }}^{2}$ for each sampled month (Table 1). **b** Lognormal density of the species abundance for each month of sampling when the abundances are confounded with sampling intensity (see Online Resource S1)

At the beginning of the ice-free period (i.e., June), the total variance of the lognormal distribution parameter *σ*^2^ was estimated to be 6*.*574 [2*.*92 − 11*.*44] (Table 1, Fig. 2). Through the summer, the estimated values of *σ*^2^ increased, while the estimates of the parameter *µ* were approximately constant (Table 1, Fig. 2). In September, unevenness was the highest (highest ${\widehat{\sigma }}^{2}$), which suggests that the community was highly uneven in this month (Fig. 2b). These results indicated a decrease in the species diversity through the summer [i.e., the abundances distribution of the community became uneven (Fig. 2b)], while the mean log abundance of the community stayed constant (Table 1, Fig. 2a).

**Table 1:** Estimated values of the parameters $\widehat{\mu }$ and ${\widehat{\sigma }}^{2}$ of the lognormal species abundance distribution and estimated values of the parameters ${\widehat{\rho }}_{\infty }$ and $\widehat{\delta }$ (i.e., strength of density regulation) of the autocorrelation function ${\rho }_{u}$ (Eq. 2)

	June	July	August	September	October
$\widehat{\mu }$	7.536 (6.344–8.683)	7.930 (6.763–9.044)	8.219 (6.789–9.614)	7.782 (6.106–9.247)	7.44 (6.023–8.866)
${\widehat{\sigma }}^{2}$	6.574 (2.92–11.44)	6.844 (2.85–11.22)	9.184 (3.891–16.747)	11.376 (5.101–21.437)	9.549 (4.598–18.170)
${\widehat{\rho }}_{\infty }$	0.30 (0.27–0.33)	0.37 (0.34–0.38)	0.41 (0.37–0.42)	0.57 (0.55–0.59)	0.56 (0.54–0.58)
$\widehat{\delta }$	1.26 (0.92–6.17)	1.89 (1.31–4.56)	1.45 (1.00–2.77)	3.32 (1.47–8.47)	2.71 (1.50–8.37)

The 95% confidence intervals obtained from bootstrapping the residuals are given in parentheses

### Component of the community dynamics

**Fig. 3:**
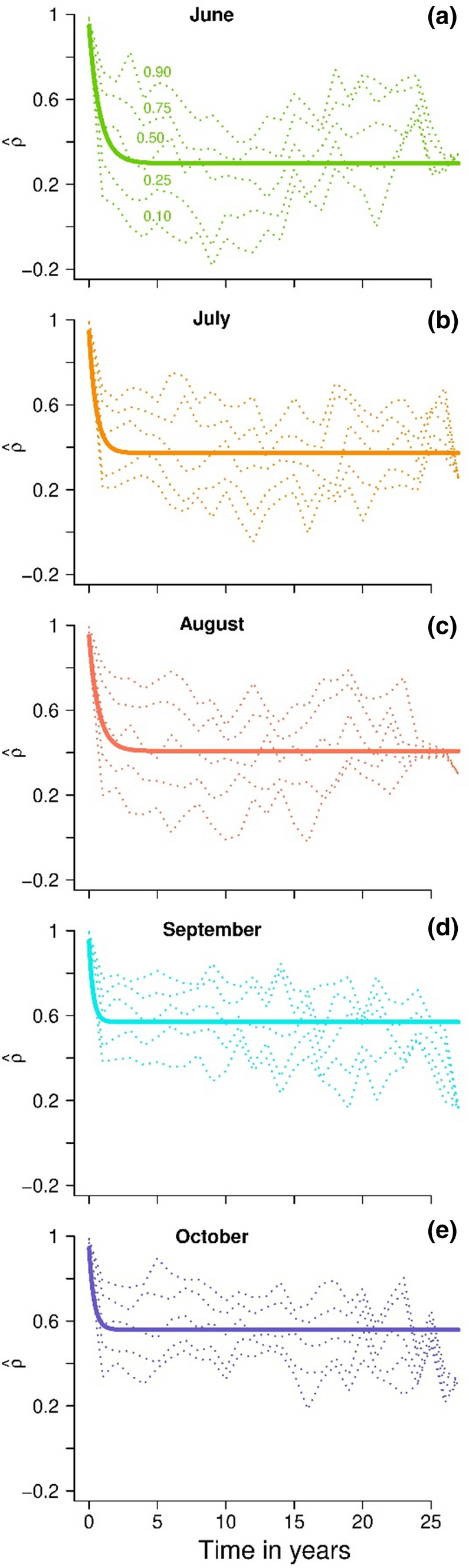
Dotted lines show estimated quantiles of the sampling distribution of the community correlation as function of yearly time lag estimated separately for each time lag from 0 to 27 for **a** June, **b** July, **c** August, **d** September, and **e** October. The solid lines show the autocorrelation temporal function (Eq. 1) fitted to all estimates of correlation by least-squares. The 95% confidence intervals of the parameters of the autocorrelation function are given in Table 1

**Fig. 4:**
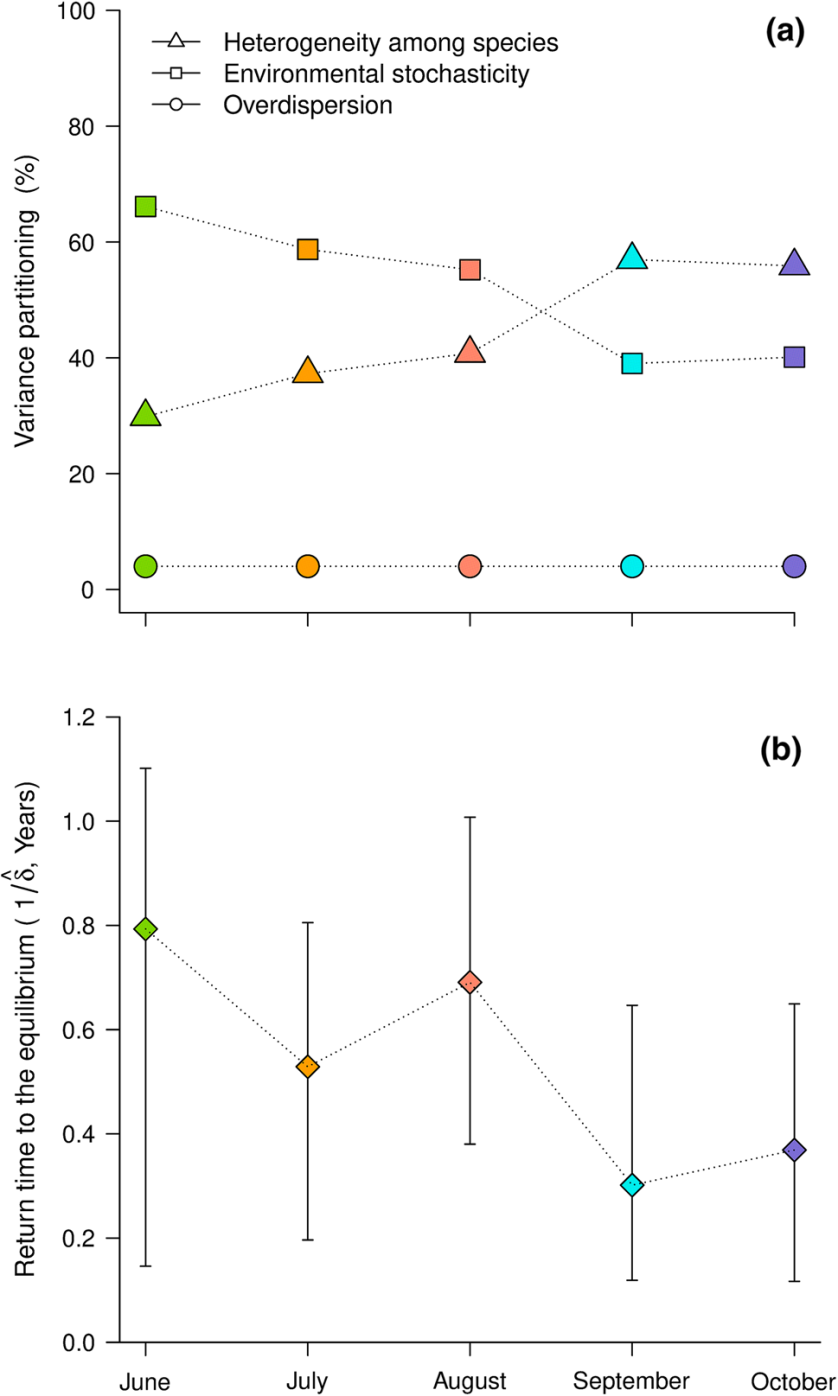
**a** Decomposition of the variance of the lognormal species abundance distribution into components generated by species-specific environmental noise, ecological heterogeneity, and sampling overdispersion for each sampling period. **b** Estimation of the return time to equilibrium (i.e., $1/\widehat{\delta }$) for each sampling period

The fitted exponential curves showed a rapidly decreasing autocorrelation (Fig. 3). The correlation *ρ*_*∞*_ significantly increased from the spring to the autumn (Table 1), while the correlations *ρ*_0_ for the parallel samples within years were stable (Table 1). We estimated for each sampling period the three components of community dynamics, environmental stochasticity, ecological heterogeneity and sampling overdispersion (Fig. 4, Table S2).

In spring, the environmental stochasticity explained 70% of the total variance and decreased until explaining only 40% at the autumn (Fig. 4). At the opposite, the ecological heterogeneity explained only 30% in spring and increase until autumn to explain more than 60% of the total variance of the lognormal species abundance distribution. The return time to equilibrium linearly decreased significantly (*p* < 0*.*0001) from the spring to the autumn (Table 1, Fig. 4). We also estimated the similarity overall months and years and fitted the temporal autocorrelation model to analyze the long-term inter-annual dynamics (Fig. S7 in Online Resource 4). The parameters ${\widehat{\rho }}_{\infty }$ was estimated to be 0.431 with 95% confidence intervals which was [0.432, 0.442] and $\widehat{\delta }$ was 9.025 (i.e., strength of density regulation) with 95% confidence intervals which was [9.025, 9.026] of the autocorrelation function *ρ*_*u*_ (Eq. 2). The 95% confidence intervals were obtained from bootstrapping the residuals. We deduced that the environmental stochasticity explained 52.8% of the variance of the log-normal distribution, the ecological heterogeneity 43% and the overdispersion 3.9%.

### Inter-annual fluctuations of species diversity

**Fig. 5:**
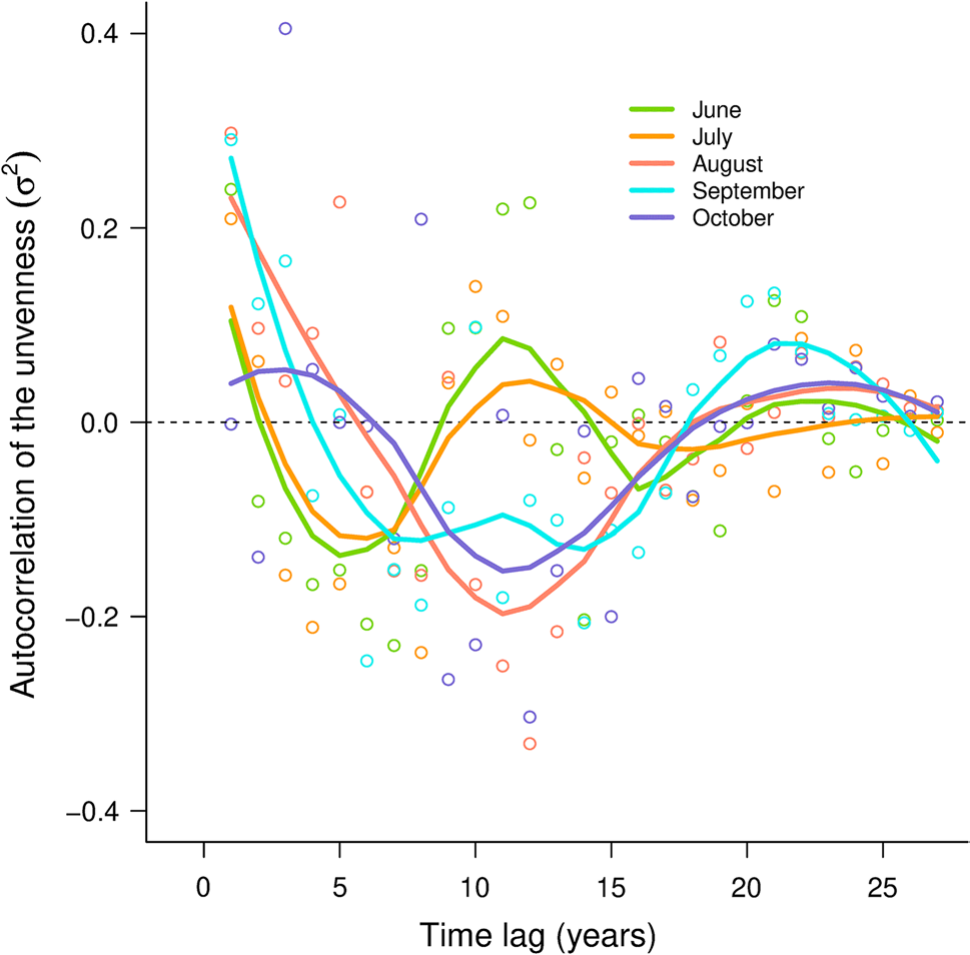
Temporal correlation patterns for unevenness (i.e., the autocorrelation of ${\sigma }^{2}$) from July to October. The dots corresponds to the temporal correlation estimates and the solid line represents the smoothed curve of the unevenness temporal autocorrelation. The 95% confidence intervals of the smoothed curves are provided in Fig. S8 in Online Resource 4

**Fig. 6:**
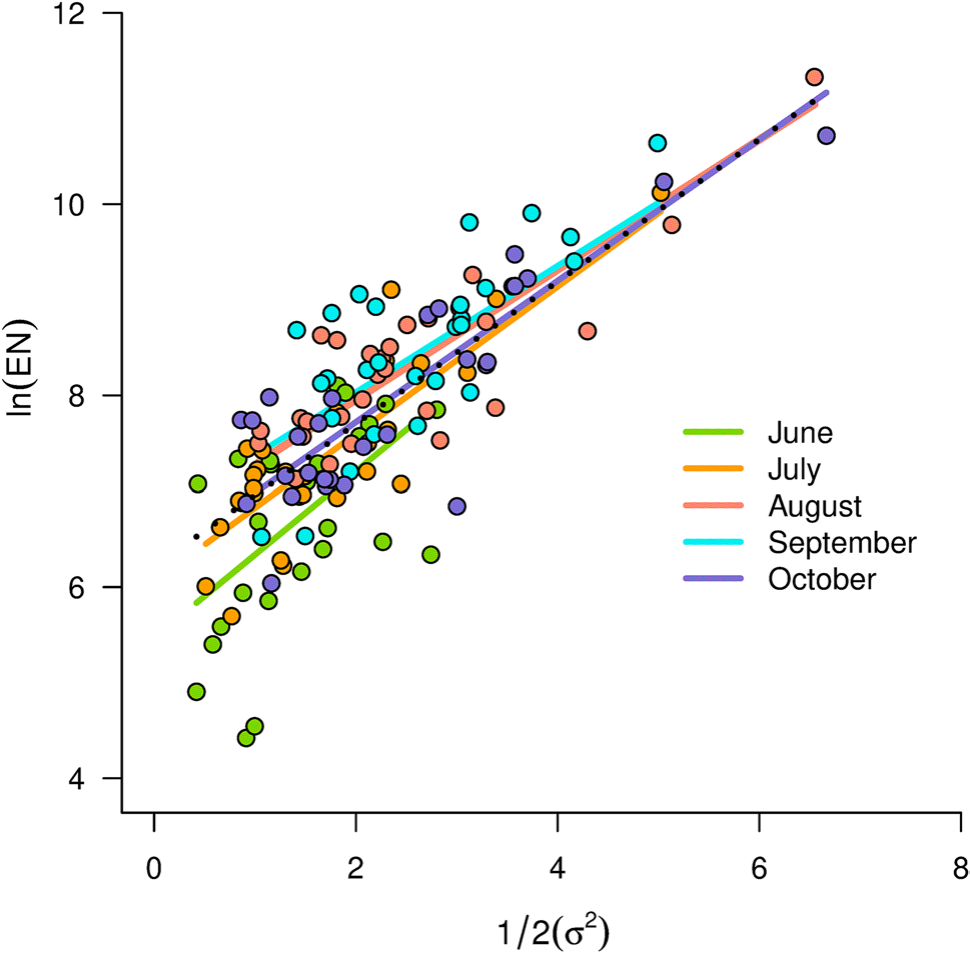
Relation between the expected number of individuals and unevenness (Table S3). The dotted line represents the model with fixed effects, while the other lines represent the contribution of the random effects (i.e., sampling period)

The unevenness (i.e., σ^*2*^) presented inter-annual correlation (Fig. 5). In spring and summer, in June and July, the unevenness presented a 10-year cycle. At the autumn, the cycle of the unevenness was about 20 years (Fig. 5). The unevenness increased when the expected number of individuals increased (Fig. 6, Table S3). If the estimates of *µ* are constant, the estimate of the slope of the relation between the expected number of individuals and the unevenness is equal to one (Fig. 6).

### Species diversity and environmental fluctuations

We used a generalized additive mixed model to analyze the unevenness (*σ*^2^) as a function of environmental fluctuations. Five environmental factors such as water temperature, water transparency, phytoplankton biomass, river run-off and length of the ice-cover period were used as co-variables (Fig. S6 in Online Resource 5) in the generalized additive mixed models (Table S4 in Online Resource 5). The sampling period (ranked from 1 to 5) was included as random effect to account for dependency between observations from the same year. We also included a yearly auto-correlation function (AR1) to account for autocorrelation of observations between years. The most parsimonious GAMM aimed to explain unevenness fluctuations included year, water temperature, and water transparency (Table S5 in Online Resource 5, *R*^2^ = 0*.*23). We constructed a GAMM model in which the co-variables were 1-month lagged (Table S6 in Online Resource 5) to identify possible effects of recurring seasonal biological events (i.e., phenology) on unevenness (Forrest and Miller-Rushing 2010) from July to October from 1990 to 2017. The final GAMM retained the 1-month lagged water temperature, 1-month lagged water transparency and 1-month lagged phytoplankton (Table S7 in Online Resource 5, *R*^2^ = 0*.*17). Unevenness increased (i.e., *σ*^2^ increased) when water temperature and water transparency increased (Fig. S9 in Online Resource 5). Unevenness increased (i.e., *σ*^2^ increased) when 1-month lagged water transparency and 1-month lagged phytoplankton increased (Fig. S10 in Online Resource 5). The GAMM models’ residuals did not present any temporal autocorrelation (Fig. S11 in Online Resource 5). The water temperature significantly increased in July from 1990 to 2017 (Table S8 and Fig. S12 in Online Resource 5).

## Discussion

One key finding of this study suggests that the zooplankton community retrieves the same distribution of abundances (i.e., community structure) every autumn. In contrast, the abundances of species forming the community differ each spring. Indeed, the estimated correlations (i.e., *ρ*), which is a measure of community similarity between each pair of years were lower in spring than in autumn. This result reflects that the structure of the community (i.e., the abundance of each species of the community) is quite different between two samples separated by a given time lag (e.g., different in spring and autumn). In spring, the environmental variability explained the largest part of the variance of the log abundances of the community’s species. The relative importance of ecological heterogeneity in comparison to environmental stochasticity shifted during the summer (i.e., ice-free period), and the ecological heterogeneity increased in autumn. The zooplankton species are organisms with fast life-histories that produce many generations within a year (Gillooly 2000). Therefore, our findings suggest that the choice of the season of sampling is crucial to study the community dynamics of organisms with short-generations times. We obtained different estimates of community dynamics components in spring and fall (Fig. 4b). Thus, failure to consider seasonal succession could lead to misleading conclusions about the community’s dynamics, particularly about the long-term change of the community structure. Depending on the sampling season, the findings regarding the identification of the prevalent factors of the community dynamics might be different (Fig. 4b). Repeated sampling through a year can be important to better understand the maintenance of species diversity, especially in the context of climate change, where the phenology of species varies among years (Forrest and Miller-Rushing 2010; Parmesan 2006; Visser and Both 2005).

The approach developed by Bellier et al. (2012, 2014) that accounts for variation of sampling intensities to improve the accuracy of the estimation of the log-normal species abundance distribution could not be applied to these data, because different species could be associated to different sampling intensities and the subsampling factors were not available. Therefore, in this study, for the most abundant species, the sampling is not Poisson, because smaller samples have been counted and multiplied with the relevant factor for those species. This makes the sampling variances larger than for the Poisson model. Using the Poisson lognormal model, the actual distributions of log abundances have been corrected for Poisson sampling. Nevertheless, this correction is too small with the actual sampling, and the variances are, therefore, slightly overestimated. However, the sampling correction for the most abundant species is relatively small and most important for the species with a complete count, so increasing it will only have a minor effect. Since the same sampling methods are used in all community samples, this minor overestimation of the variances of log abundances only has negligible effects on the results of our analyses.

In this study, the approximate fraction of the species revealed by the pooled sample was relatively high. Lake Atnsjøen is an oligotrophic lake, where the density of species can be relatively low (Lampert and Sommer 2007). Therefore, the sampling could represent well the species diversity of the lake. This could be because some species were determined only until the genus (Table S1). Nevertheless, here, the approximate fraction of the species revealed by the sample were only estimated for the pooled sample and not analyzed in space and time.

Environmental variability highly influenced the composition of the initial zooplankton community in early spring. Indeed, in spring, many of the zooplankton species hatch from resting eggs, and the populations of individual species are initially low. Hence, environmental variability might affect the succession of species and produce large yearly fluctuations in species abundances. During the winter, the weather can influence resting eggs’ contribution to population development (Gillooly et al. 2002; Sommer et al. 2012). In turn, this might affect the structure of the abundances of the community differently each year, because the number of individuals coming from overwintering females and the time of ontogenetic development might vary (Gillooly et al. 2002). In lake ecosystems, the phytoplankton spring bloom is typically short-lived because of grazing, nutrient depletion, and cell sinking, resulting in a crash of phytoplankton, which produces a clear water phase (Sommer et al. 1986, 2012). During the clear phase (i.e., summer and autumn), regulating population dynamics mechanisms are more complicated as biotic factors such as competition and predation become more critical. In Lake Atnsjøen, the main predators of the zooplankton community are the brown trout (*Salmona trutta*) and Arctic charr (*Salvelinus alpinus*) feed on the most common species of cladoceran *Bosmina longispina*, *Daphnia longispina* and *Holopedium gibbum*. This can cause a substantial decrease in abundances in these zooplankton species through the summer, which can alter the zooplankton community structure (Hessen et al. 2006; Saksgard and Hesthagen 2004). In an experimental study, copepods’ response to fish predation depended on the seasonal successional stage of the initial community because of changes in their stage structure (Beisner and Peres-Neto 2009). In a natural lake experiment, the introduction of a top predator modified the zooplankton community structure (Elser and Carpenter 1988). This outcome suggests a possible top-down regulation of the ecological system.

Furthermore, it is well demonstrated in natural systems that predators can strongly influence prey community structure (Brooks and Dodson 1965; Carpenter et al. 1985; Sih et al. 1985). Different predator species generate different prey communities due to differences in morphology and foraging behavior (McPeek 1998; Post et al. 2008). Our results indicated that the ecological heterogeneity prevailed in the autumn, and the community size was larger (Fig. 3). A predator can either increase or reduce local prey diversity, depending on how the various organisms in the community interact (Holt 1977). This result suggests that in the autumn, competitive processes (i.e., the community contains more individuals) might dominate over stochastic processes, leading to a more predictable community structure (Chase et al. 2002). These results also indicate that, during the spring, the ecosystem of Lake Atnsjøen might be controlled by bottom-up processes (i.e., driven by the presence or absence of the producers in the ecosystem) as phytoplankton spring bloom is associated with different community structures each year. The fish predation pressure on zooplankton increases during summer and early autumn (i.e., September). The fishes (i.e., charr and trout) eat primarily cladocerans, which are herbivorous zooplankton, and strong competitors of rotifers (also herbivorous). However, when the number of cladocerans decreases, because they are eaten, it releases the rotifers from their competitor. This indirect effect of increased top-down control as summer proceeds might explain the high abundance of rotifers in August and September. Thus, our results suggest an alternation of bottom-up and top-down regulation over the growing season.

Although the expected log abundance of a species in the community (i.e., estimates of *μ*) was constant, the unevenness (*σ*^2^) increased through the ice-free period. Accordingly, estimates of ecological heterogeneity (variation among species-specific growth rates leading to variation among species in mean abundance) showed a similar increase during the season. In addition, estimates indicated that density regulation was strongest in autumn, leading to a shorter return time towards equilibrium in case of perturbations (Fig. S13). Larger interspecific variation in equilibrium abundances, as well as short return times towards these equilibriums, diminish the effects of species-specific environmental effects dominating during spring and summer (Fig. S14). This causes relative abundances in the community during autumn months to be highly correlated among years, i.e., the community exhibits high similarity. In contrast, spring and summer months with dynamics characterized by higher relative contribution from species-specific environmental noise, less ecological heterogeneity, and weaker density dependence leads to less similarity of relative abundances across years during these months. A more uneven community indicates an increasing inequality in the distribution of species ecological traits (Cornwell and Ackerly 2009; Hillebrand et al. 2008), and differences in species ecological traits lead to differences in dynamics, which also implies that parameters defining the dynamics vary among species in the community (Engen 2007). Our results indicate that the community could reach equilibrium faster when the community was dominated by ecological heterogeneity rather than environmental stochasticity. An ecosystem can be more or less stable, depending on how species belonging to a community recover from low density (Chesson 2000; May 1973).

Higher estimates of *σ*^2^ implies reduced evenness in the community, i.e., larger variation in relative abundance among species. Our results show that the cycles in unevenness have a longer time scale than the generational time scale of the species in the community. Indeed, the interpretation of annual cycles in *σ*^2^ depends on the mean generation time of species in the community being sufficiently short for community composition to respond significantly to seasonal cycles. The most common species were the rotifer, such as *Polyartha vulgaris*, *Kelicotia longispina* and *Keratella hienalis*, characterized by a short, complex life cycle. Populations develop via parthenogenesis during favorable conditions; and then by sexual reproduction with resting-egg production occurring during later, unfavorable conditions in late summer and autumn (Hairston et al. 2000). For the copepods, such as *Cyclops scutifer* and *Bosmina longispina*, the development may take from less than 1 week to as long as 1 year, and the life span of a copepod ranging from 6 months to 3 years (Elgmork 1981, 2004). Some other less common species, such as *Heterocope saliens,* have longer life-cycle (i.e., 1 year) (Larsson 1978). In autumn, the unevenness’s long-term cycle was longer (20 years) than in spring (10 years), suggesting that the community responds to long-term environmental drivers. Cycling unevenness was also related to different species’ life-cycle in a tropical butterfly community (Grøtan et al. 2012). Overall, our results show that in spring, the community is characterized by high species diversity, slow return time to equilibrium, and high temporal turnover of species mainly driven by environmental stochasticity. In contrast, in the autumn, the community is characterized by low species diversity (i.e., lower evenness), fast return time to equilibrium, low temporal turnover of species mainly driven by the ecological heterogeneity. Therefore, our findings show that ecological heterogeneity is vital to maintain ecosystem function stability. The stability of an ecosystem is important, because it reinforces its relative ability to recover from perturbations (Tilman et al. 2006). Moreover, our results also reflect the prevalent operation of non-neutral mechanisms in natural communities. During the months when the community structure was rearranged, such as September and October, the community dynamics were driven by ecological heterogeneity and long-term stability in the structure (i.e., 10-year cycle in *σ*^2^). These months are most likely key months to monitor long-term changes in community structure as noise from early summer is filtered away, and it is easier to track long-term changes. Persistent changes in community structure might result from factors affecting the ecological heterogeneity in autumn.

Our results suggest that environmental variability drives the cycling in unevenness, as they were no temporal autocorrelation in the GAMM models’ residuals. Thus, the environmental variables can at least serve as proxies for the environmental drivers. The unevenness of the zooplankton community increased with increasing water temperature and 1-month lagged water temperature (Figs. S9, S10) throughout the study. In other words, our results indicate that zooplankton diversity is inversely proportional to temperature, while zooplankton abundance is directly proportional to temperature. These results suggest that long-term variability in temperature affects species diversity. The number of individuals of given zooplankton species is closely related to temperature; when the temperature increased, the total number of individuals might be dominated by rotifers with their growth rates related to temperature (Fig. S14). Moreover, our results suggest that when the temperature increases, the increasing number of individuals of given species, such as *Cyclops scutifer* and *Bosmina longispina*, might generate stronger competition among species. Some species such as *Cyclops scutifer*, *Bosmina longispina* and *Daphnia longispina* might become much more abundant than the others (Fig. S13). Some zooplankton species can present a peak at different times, which can affect the interactions among species, such as competition (Pomati et al. 2012; Shurin et al. 2010). In Lake Atnsjøen, water temperature affects growth rates of the parthenogenetic species *Keratella* and *Daphnia* similarly. For the slower developing copepods, such as *Cyclops scutifer*, temperature change might require a longer time to change the dynamics (Stemberger and Gilbert 1985). Our results are consistent with a study showing that zooplankton species responded to the long-term warming of water temperature in boreal lakes in Alaska (Carter and Schindler 2012). In addition, the 1-month lagged temperature and 1-month lagged phytoplankton abundance is associated with an increase in unevenness of the zooplankton community (Fig. S10). An increase in the unevenness of zooplankton is coherent, because it is like a predator–prey relationship that involves a delay (Holling 1965). The emergence time of new generations of zooplankton species (eggs, nauplii) can vary with the intensity of phytoplankton production (Sommer and Lengfellner 2008). For plant communities, climate plays an essential role in inter-annual variability of species population growth rate, which allows for differentiation in species dynamics (Adler et al. 2006). Our results suggest that warming temperature can lead to variability in the abundance of species, which affects community structure over time. The modifications of the community structure over time can have cascading effects through the food web and affect the functioning of the ecosystem (Carpenter et al. 1985; Yachi and Loreau 1999).

Our findings suggest evidence in a natural system that seasonal variation in community dynamics can cause seasonal variation in community structure, which contribute explicitly to better understand how species co-exist in a fluctuating environment. This is important, because biological diversity is essential to the proper functioning of the ecological systems (Cardinale 2012; Hooper et al. 2012). Overall, our results show that temperature acted on the diversity of species in the long term; increasing temperature could reduce the high diversity observed in spring and, consequently, affects the stability of trophic relationships.

## Supplementary Information

Below is the link to the electronic supplementary material.

Supplementary file1 (PDF 3137 KB)

## Data Availability

The data sets analysed during the current study are available from the corresponding author on reasonable request.

## Code availability

The code developed during this study might be available from the corresponding author on reasonable request.
