# Supplementary material for: Seasonal diversity dynamics of a boreal zooplankton community under climate impact
Source: Oecologia. 2022 Apr 26;199(1):139–52. doi: 10.1007/s00442-022-05165-0 (PMC9120095; doi:10.1007/s00442-022-05165-0)
Supplement: Supplementary file 1 — Supplementary file1 (PDF 3137 KB) [file 442_2022_5165_MOESM1_ESM.pdf]

Electronic Supplementary Information

for

Seasonal diversity dynamics of a boreal zooplankton community under climate impact

Edwige Bellier[1], Steinar Engen[2] and Thomas Correll Jensen[3]

[1]Department of Arctic and Marine Biology, UiT The Arctic University of Norway NO-9037, Tromsø, Norway.
[2]Centre for Biodiversity Dynamics, Department of Mathematical Science, Norwegian University for Science and Technology, N-7491 Trondheim, Norway.
[3]Norwegian Institute for Nature Research, Sognsveien 68, NO-0855 Oslo, Norway.

Corresponding author: Edwige Bellier, present address: Department of Natural Resources Science, University of Rhode Island, Kingston, 02881, RI, USA.
Email: edwbellier@gmail.com
Phone number: (+1) 401 874 2026

Online Resource 1

The bivariate lognormal species abundance model

The model is based on a general stochastic theory, with speciation/colonization, extinction and environmental populations fluctuations (Engen and Lande, 1996). For data sets collected over a relatively short time period, the model can be approximated by a stationary model with a constant given number of species ignoring possible changes in species composition during the sampling period (Engen et al., 2011). Each species is then described by an Ornstein-Uhlenbeck (OU) process (Karlin and Taylor, 1981) in the sense that the dynamics of log abundance X_i of species number i is given by,

$$dX_i = (r_i - \delta X_i)dt + \sigma_s dB_i(t) + \sigma_c dB_c(t) \tag{S1}$$

where t denotes time. Here r_i is the stochastic growth rate of species i in the absence of density regulation (small population sizes) and δ is the strength of density-regulation, giving carrying capacity $K_i = e^{(r_i/\delta)}$ varying among species. The functions $B_i(t)$ and $B_c(t)$ denote OU brownian motions in time t with the properties $EdB(t) = 0$ and $EdB(t)^2 = dt$. The $dB_i(t)$ are noise components that are independent among species, while $dB_c(t)$ represents a noise that is common for all species in the community, that is, similarly affecting all species abundances. The common noise may in general have a given correlation ρ_{sc} with each species specific term that is $EdB_i(t)dB_c(t) = \rho_{sc}dt$. The noise for a single species can alternatively be written as $\sigma_e dA_i(t) = \sigma_s dB_i(t) + \sigma_c dB_c t$ where $\sigma_e^2 = \sigma_s^2 + \sigma_c^2 + 2\rho_{sc}\sigma_s\sigma_c$ is the total environmental variance for each species single species considered separately and the $A_i(t)$ are dependent Brownian motions. The stationary variance for the Ornstein-Uhlenbeck process is $\sigma_e^2/2(\delta)$ and the temporal autocorrelation is the exponential function $e^{-\delta t}$. The variances in the yearly change in log population size for a population of size N is generally $\sigma_e^2 + \sigma_d^2/N$, where σ_e^2 and σ_d^2 are the environmental and demographic variance (Engen etal. 1998; Lande etal. 2003). Here, we analyse communities of small organisms with large individual numbers from which only tiny fractions are sampled. So, even if the demographic variance may be of some orders larger than the environmental variance say 10-100 times as large, the temporal variation in variance, due to temporal variation with varying N, can be ignored. On the other hand, demographic stochasticity may lead to some minor spatial variation in abundance. When sampling is done at a given spatial position, such an effect may appear as confounded with the sampling overdispersion. Assuming constant species number during the period of data collection, each X_i will be normally distributed with mean value r_i/δ and variance $\sigma_e^2/(2\delta)$. We assume that the r_i are themselves normally distributed in the community with mean, say r_0, and variance σ_r^2, the log abundances $(X_1, X_2, X_3, \ldots, X_n)$, then is a sample from the normal distribution with mean r_0/δ and variance $\sigma_e^2/2\delta + \sigma_r^2/\delta^2$. The last term is the variance in log carrying capacities among species which are lognormally distributed. This distribution which is unaffected by the stochasticity of each species, may be a consequence of species dividing the niche space between them over long time periods. Further, Engen et al. (2002) have shown that two sets of log abundances $(X_1, X_2, X_3, \ldots, X_n)$ and $(Y_1, Y_2, Y_3, \ldots, Y_n)$ for the same community recorded at time differences t, can be considered as pairs of log abundances $(X_1, Y_1), (X_2, Y_2), \ldots, (X_n, Y_n)$ that are independent observations from the bivariate normal distribution with variance $\sigma_s^2/(2\delta) + \sigma_r^2/\delta^2$ and covariances $\sigma_s^2/(2\delta) + \sigma_r^2/\delta^2$. The specific noise σ_s^2 represents stochastic components that are independent among species. In addition to variation in carrying capacity that may have be an aspect of long-turn sub-division of the niche space, these terms, which are also generated by species-specific traits varying among species, may be related to niche aspect describing temporal fluctuations in the sub-division.

Online Resource 2

The Poisson lognormal species abundance distribution

To describe integer numbers of individuals in a sample from a community, we first follow Fisher et al. (1943) and Bulmer (1974) by assuming that the number of individuals of a given species observed in a sample is Poisson distributed with mean proportional to the abundance of the species in the community. The number of individuals, N, sampled for a given species with log abundance x in the community is then Poisson distributed with mean $\nu e^x = e^{x+\ln\nu}$, where the parameter ν expresses the sampling intensity. Assuming that abundances of different species in the community are lognormally distributed, x is then normally distributed among species with mean μ and variance σ^2, whereas the log of the Poisson mean, $x + \ln\nu$, is normal with mean $\mu + \ln\nu$ and the same variance σ^2. The number of individuals N sampled from a random species in the community then constitutes a sample from the Poisson lognormal distribution with parameters $(\mu + \ln\nu,\ \sigma^2)$. The parameter μ in the underlying abundance distribution cannot be estimated unless the sampling intensity is known or can be estimated in some way. The sampling intensity affects the expected number of individuals in the sample, but has no influence on the form of the distribution as the variance parameter σ^2 is unaffected by changing sampling intensities (Engen et al. 2008, Connolly etal. 2009). Following Fisher et al. (1943) and Bulmer (1974), we only consider the numbers of individuals of species that are represented in the sample. Thus, there is an unknown number of species that are present in the community but are absent from a sample from the community. The observed numbers of individuals of the different species therefore follow a zero-truncated distribution because the number of species with a count of zero is unknown. The expected fraction of unobserved species given a set of parameters in the Poisson log-normal distribution and a certain sampling is $q(0; \mu + \ln\nu, \sigma^2)$, and the zero-truncated distribution is,

$$\frac{q(n; \mu + \ln\nu, \sigma^2)}{1 - q(0; \mu + \ln\nu, \sigma^2)} \tag{S2}$$

defined for $n = 1, 2, \ldots$ The maximum likelihood estimation of the parameters of this distribution was first derived by Bulmer (1974).

The bivariate lognormal species abundance distribution

If we jointly consider two communities (sampled from different locations and/or times), each species will be represented by a realization of a of a bivariate random variable expressing its abundance in the two communities (Engen etal. 2002, 2008). Still assuming a lognormal species abundance distribution as the marginal distribution in each community, the log abundances in two communities will follow a bivariate normal distribution with parameters $(\mu_1; \sigma_1^2, \mu_2; \sigma_2^2; \rho)$. The parameters $(\mu_1; \sigma_1^2)$ and $(\mu_2; \sigma_2^2)$ represent the marginal lognormal distributions in the two communities. A high positive correlations means that a given species is relatively common (or rare) in the first community will tend to have similar relative abundance also in the second community. Assuming Poisson sampling leads to a bivariate distribution of the number of individuals of a species in the two samples (N_1, N_2) conditional on presence in at least one of them. Species that are present in both communities but absent in both samples need to be accounted for by using a truncated distribution. The zero-truncated bivariate Poisson-lognormal distribution takes the form

$$\frac{q(n_1, n_2; \mu_1 + \ln \nu_1, \sigma_1^2, \mu_2 + \ln \nu_2, \sigma_2^2, \rho))}{1 - q(0, 0; \mu_1 + \ln \nu_1, \sigma_1^2, \mu_2 + \ln \nu_2, \sigma_2^2, \rho)}, \tag{S3}$$

where the function q here is refined for the two dimensional case. The parameters σ_1^2, σ_2^2 and ρ can be estimated without any knowledge about the unknown sampling intensities ν_1 and ν_2 that may differ among different samples. Estimates of μ_1 and μ_2 can only be found if sampling intensities are known. This approach can be generalized to deal with over-dispersion relative to the Poisson. Following Engen et al. (2002), such over-dispersion is handled most efficiently by assuming that the number of representatives of a species with log abundance X is Poisson-distributed with parameter $\nu V e^x$, where $\ln V$ is itself a normal variate with mean $\theta^2/2$ and variance θ^2, so that V is a lognormal variate with mean 1 taking independent values for each single sampling of a species. The distribution of V expressing over-dispersion may include a component due to minor spatial variation generated by demographic stochasticity. With these assumptions the bivariate discrete distribution is still the bivariate Poisson lognormal with the same parameter μ_x, μ_y and c_{xy}, but with variance parameters $\sigma_x^2 + \theta^2$ and $\sigma_y^2 + \theta^2$.

The R-package `poilog` (Grøtan and Engen, 2008) was used for estimating the parameters in the univariate and the bivariate Poisson lognormal distribution.

Online Resource 3

Summary of the modelling approach

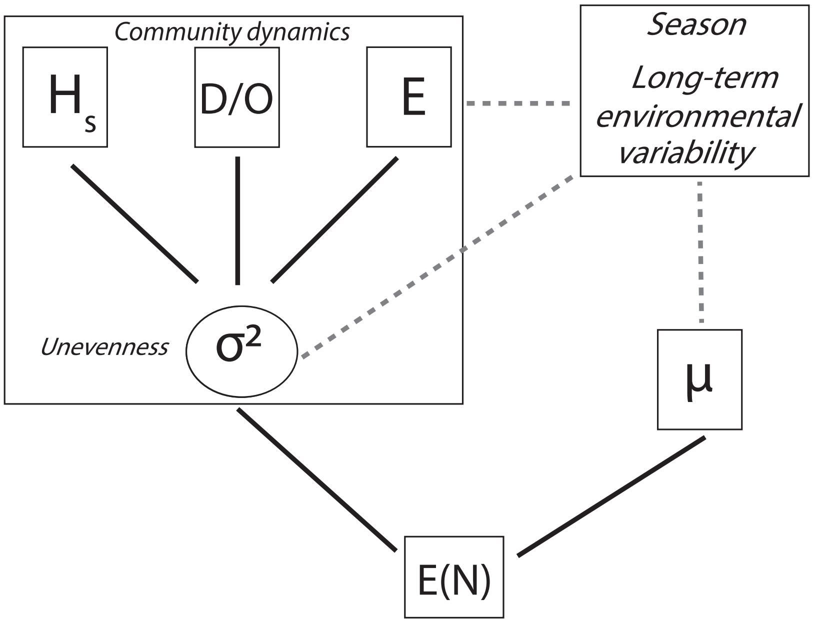

Figure S1: Schematic representation of the modelling approach to analyse how environmental variability can affect the maintenance of the species diversity through its effect on community dynamics. The key parameters is the variance of the lognormal distribution (σ^2) which enables to quantify the unevenness and can be partitioned to estimate of the components of the community dynamics (see Eq. 5). "H_s" represents the functional diversity, "$D/0$" represents the overdispersion and the demographic stochasticity, "E" represents the environmental stochasticity. The total number of individuals $E(N)$ can be estimated from the mean parameter μ and the variance parameter σ^2 of the lognormal distribution (see Eq. 6). The black line represents the connection between the parameters of the lognormal species abundance distribution and the community dynamics characteristics; the dotted grey line represents the influence of seasonality and the long-term environmental variability. The analysis of the effects of environmental variability on species diversity (i.e., unevenness σ^2) is realized by using generalized additive mixed models see section "species diversity and environmental fluctuations" in the main text and Online Resource 5.

Online Resource 4

Zooplankton community in Lake Atnsjøen and environmental variables

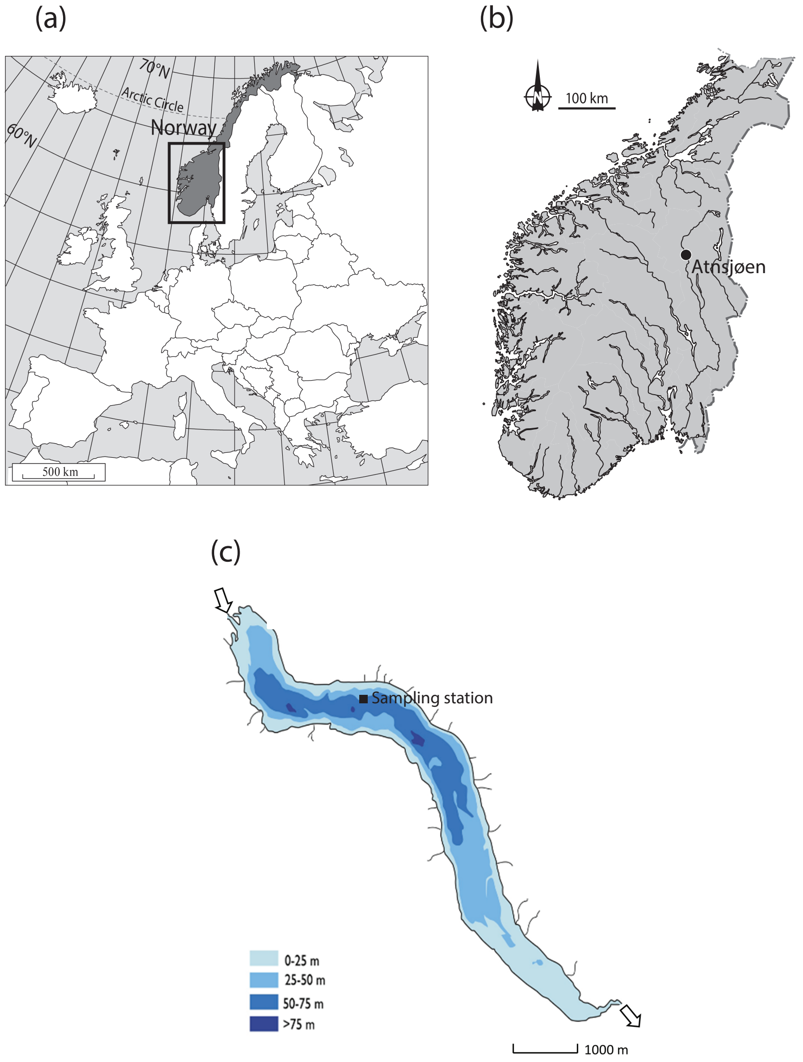

Figure S2: (a) Localisation of South-Norway (b) Localisation of Lake Atnsjøen from (c) Localisation of the sampling station in Lake Atnsjøen.

Table S1: Species names and classfication of the community of zooplankton of Lake Atnsjøen.

Species name	Classification
Keratella cochlearis	Rotifera
Keratella hiemalis	Rotifera
Keratella serrulata	Rotifera
Kelicotia longispina	Rotifera
Lecane sp.	Rotifera
Ascomorpha sp.	Rotifera
Polyarthra vulgaris	Rotifera
Polyarthra remata	Rotifera
Polyarthra dolichoptera	Rotifera
Asplanchna sp.	Rotifera
Conochilus unicornis	Rotifera
Synchaeta pectinata	Rotifera
Synchaeta oblonga	Rotifera
Collotheca mutabilis	Rotifera
Trichocerca sp.	Rotifera
Brachionus rubens	Rotifera
Rotaria sp.	Rotifera
Notholca sp.	Rotifera
Euchlanis sp.	Rotifera
Cephalodella sp.	Rotifera
Cyclops scutifer	Copepoda
Bosmina longispina	Cladocera
Daphnia longispina	Cladocera
Holopedium gibberum	Cladocera
Polyphemus pediculus	Cladocera
Bythotrephes longimanus	Cladocera
Alona sp.	Cladocera
Arctodiaptomus laticipes	Copepoda
Acanthocyclops vernalis	Copepoda
Chydorus sp.	Cladocera
Diaphanosoma brachyurum	Cladocera
Heterocope saliens	Copepoda

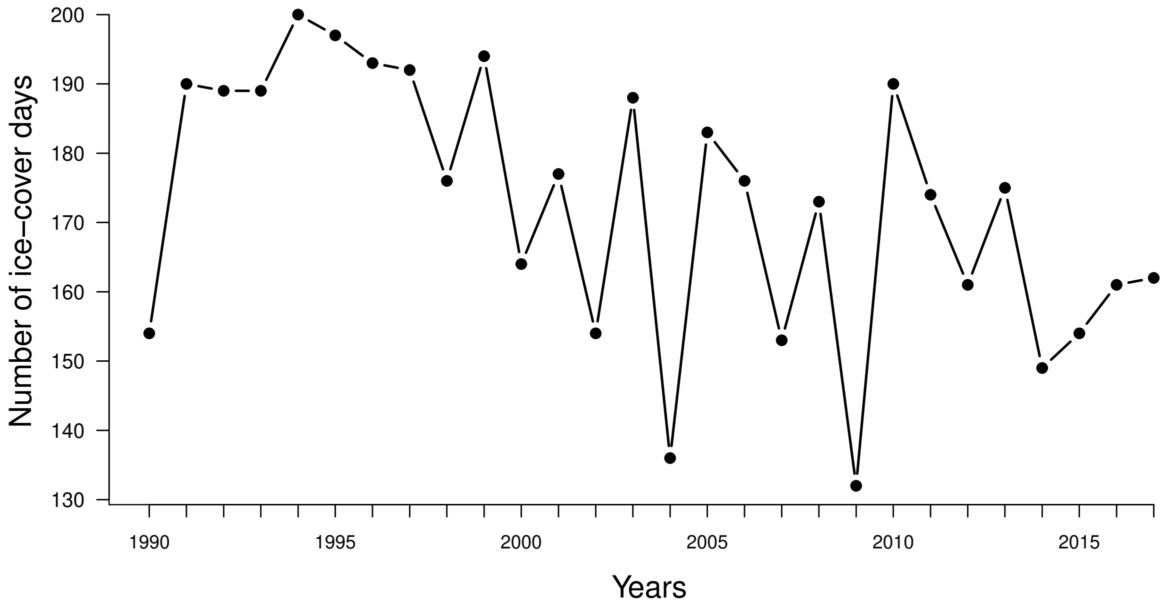

Figure S3: Number of days where Lake Atnsjøen was covered by ice during each winter from 1990 to 2017.

Most common species

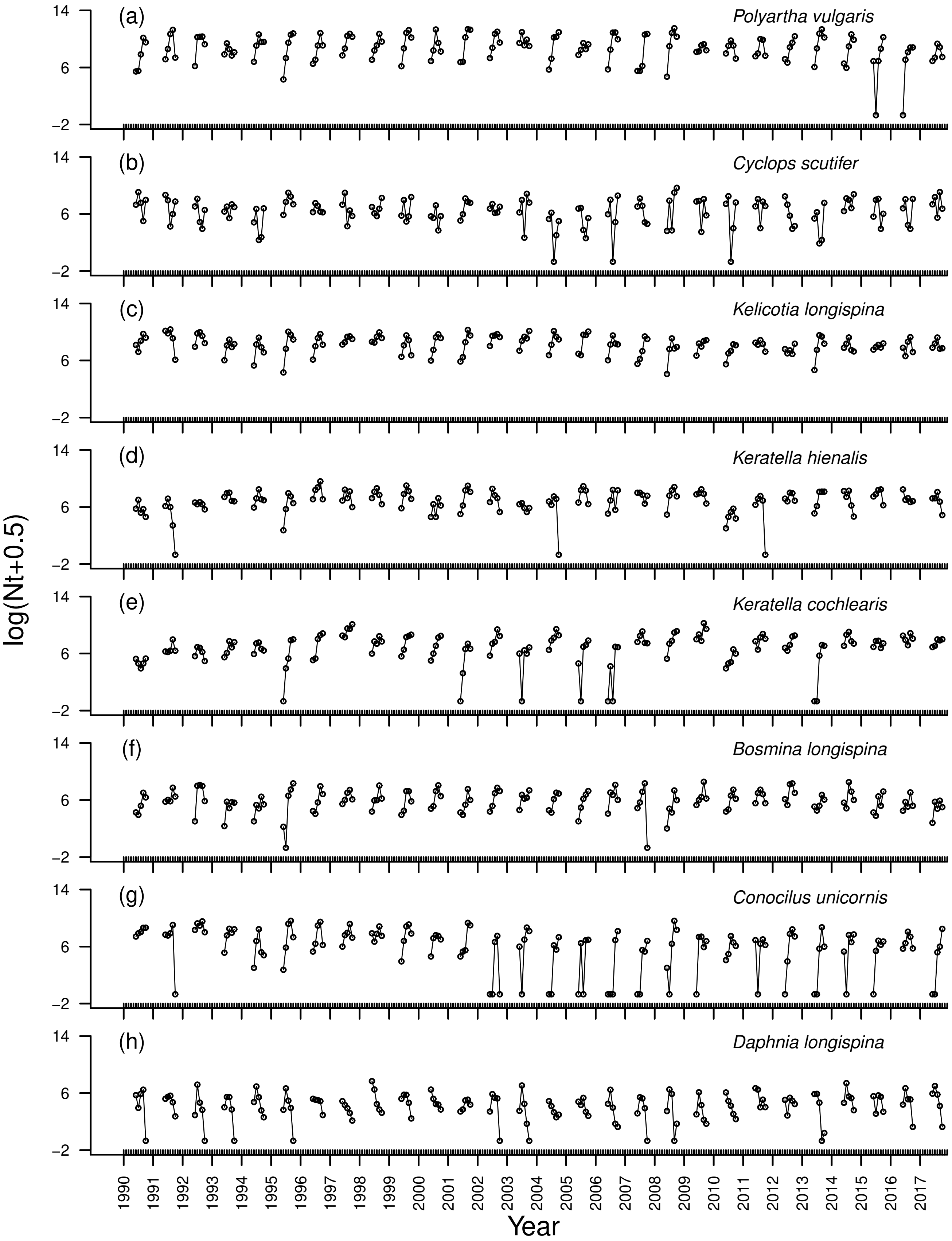

Figure S4: Time series of the eight most common species of freshwater zooplankton collected during during the ice-free period from 1990-2017 in the Lake Atnsjøen (Norway).

Species diversity and environmental variables

We chose a set of 5 covariables selected regarding their consistency of measures on every dates of the time-series using the same method and their potential effects on species diversity. Temperature is an important determinant of population growth rate and might affect generation time of zooplankton species (Gillooly, 2000). In Lake Atnsjøen water transparency is partly determined by the phytoplankton biomass (Brettum and Halvorsen, 2004) and by the input of dissolved organic carbon (DOC) which can affect zooplankton communities. The transparency can also affect the predation pressure of fish on the zooplankton, because the planktivorous fishes depend on good visibility to detect their zooplankton prey (Mazumder et al., 1990). Phytoplankton biomass is a proxy of primary production that can affect zooplankton abundance and diversity (Hessen et al., 2006). River run-off can have indirect effect on zooplankton community composition by disturbing the chemical and physical properties of the Lake and increasing the zooplankton mortality through wash out (Tvede, 2004). The duration of ice-cover is important for lake ecological systems as it influences physical, chemical and biological processes (Walsh et al., 1998).

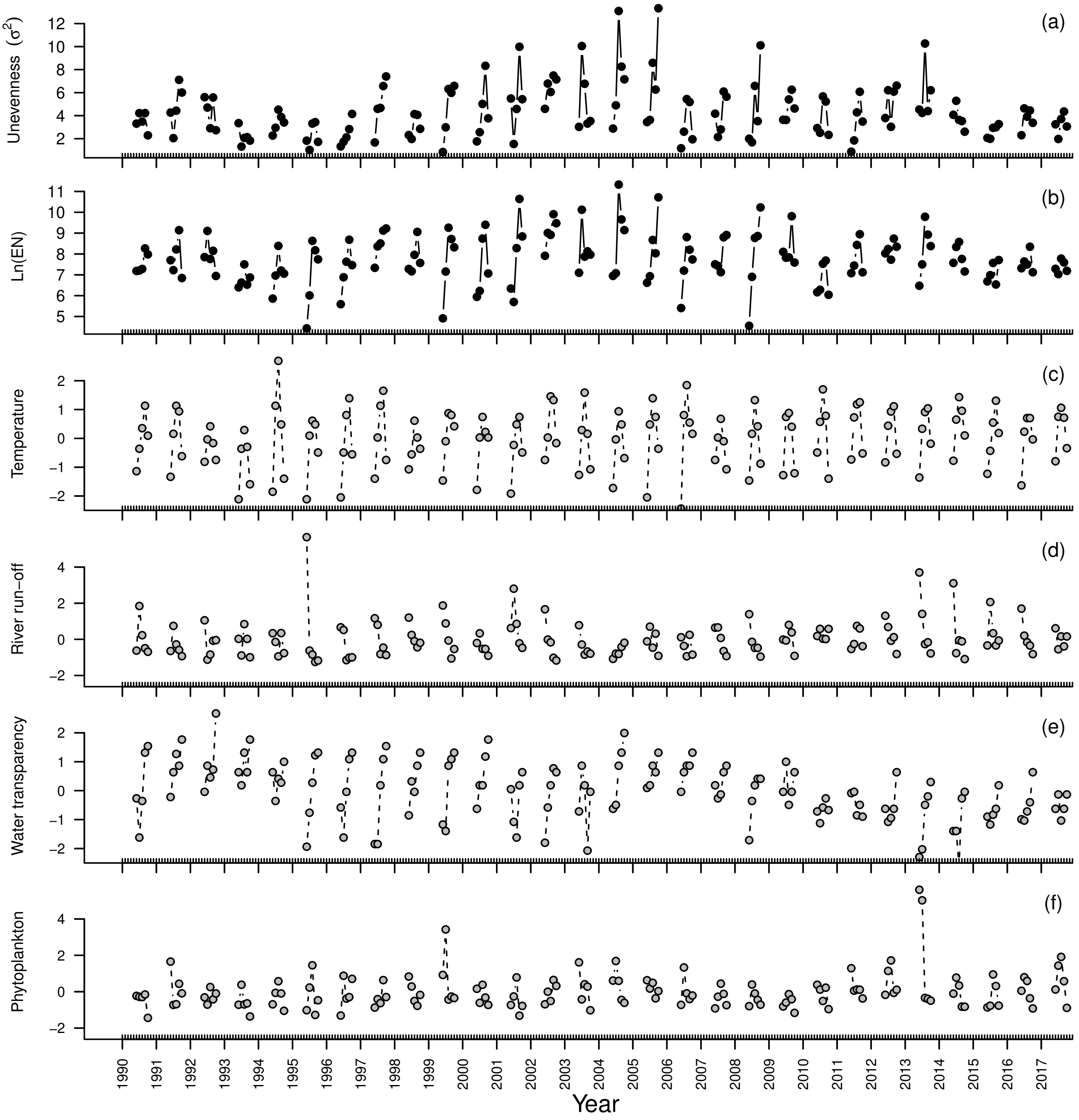

Figure S5: Long-term time series from 1990 to 2017 of the (a) estimated unevenness (σ^2), (b) total number of expected number individuals, (c-f) time series of the environmental variables.

Species abundance distribution and components of the community dynamics

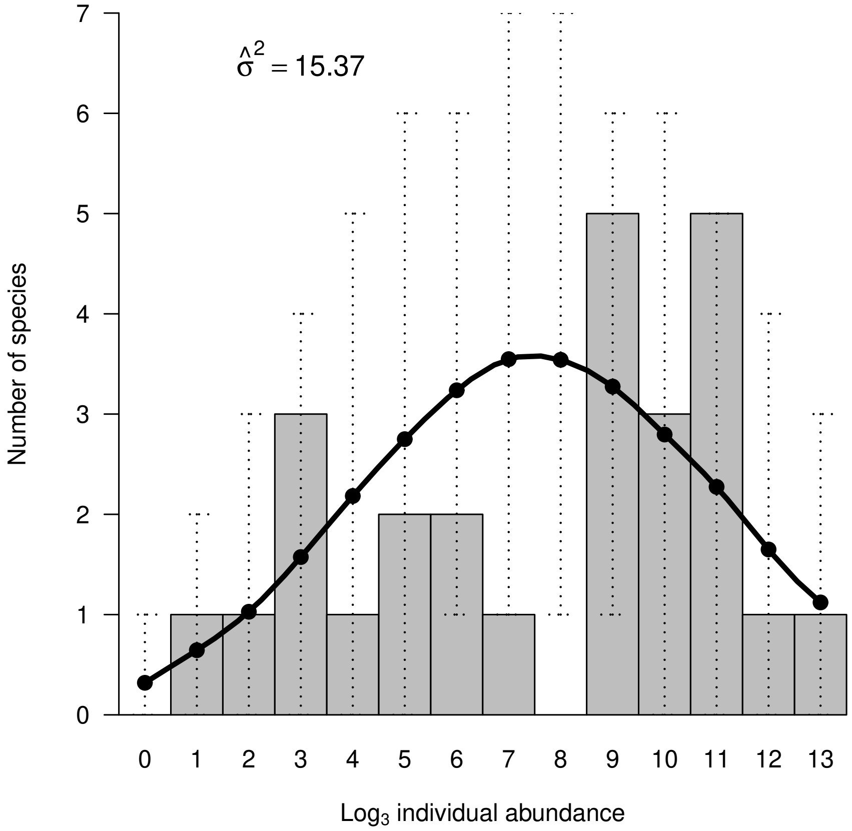

Figure S6: Species abundance distribution of the pooled zooplankton community of Lake Atnsjøen from 1990 to 2017. Large dots: expected Poisson–lognormal distribution, simulated by first sampling species expected abundances from the fitted lognormal and then Poisson sampling numbers of individuals within species; error bars are 95 % confidence intervals from repeated simulations. Species abundance data are displayed in discrete bins on a logbase 3 scale, with edges at $3^j/2$ for $j = 0, 1, 2, 3, ...$, containing $1, 2-4, 5-13, 14-40, ...$ individuals per species (Williams, 1964; Lande et al., 2003). The parameters of the pooled community are estimated from the lognormal distribution using `poilog` (Grøtan and Engen, 2008). The smoothed curve represents the lognormal distribution fitted using `poilog`.

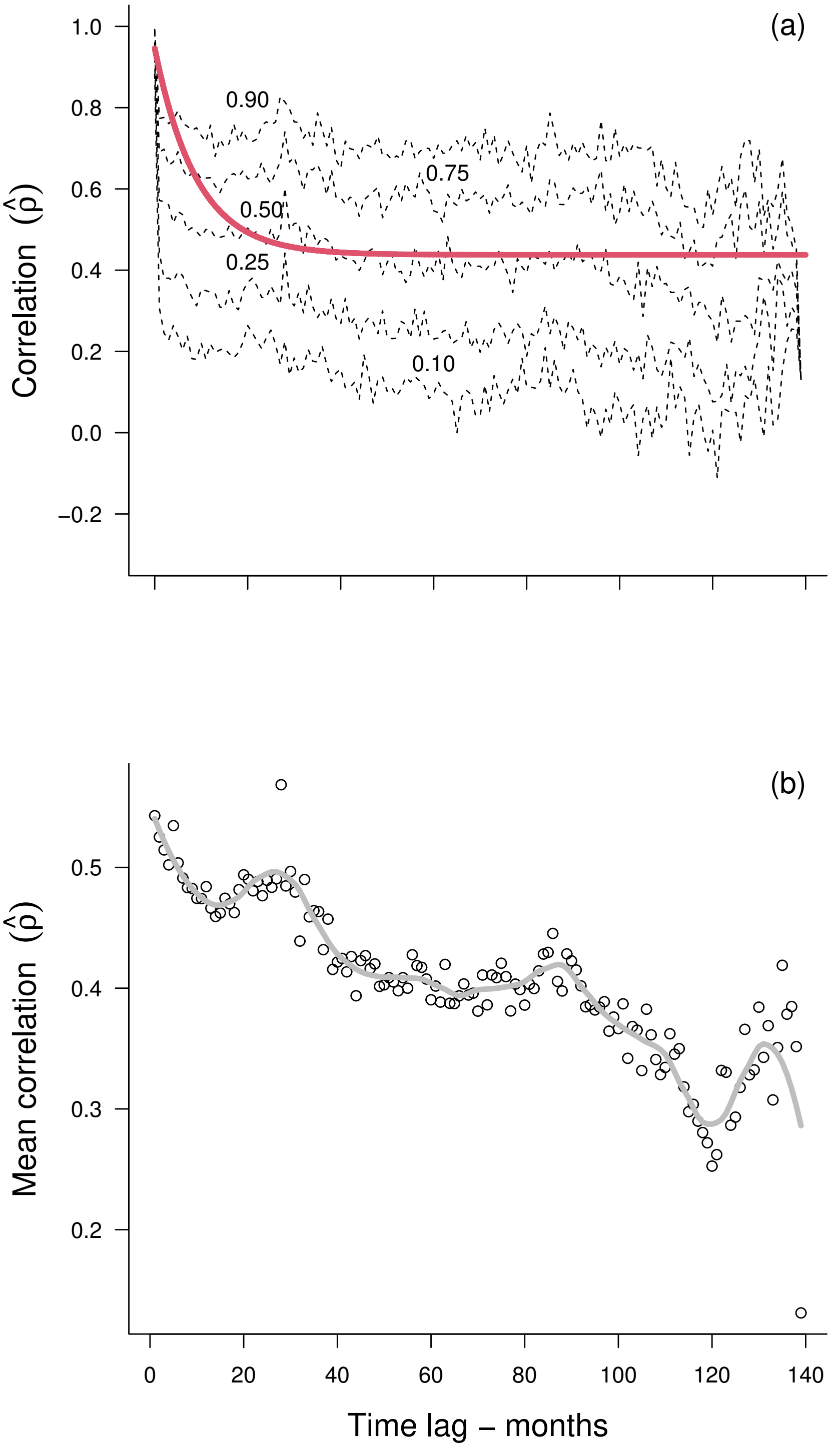

Figure S7: (a) Estimated quantiles of the sampling distribution of the community composition correlation from June 1990 to October 2017 (i.e., 140 months) as a function of the time distance computed separately for each distance from 0 to 140 (i.e., dotted lines); the solid red line shows the exponential function (Eq. 1) fitted to all estimates of correlation by least-squares. (b) Mean correlation as a function of monthly time lag for all the period of study from June 1990 to October 2017 (i.e., 140 months); the solid grey line represents the smoothed curve of the mean correlation.

Estimation of the sampling variance of the expected number of individuals

We deduce var[ln($E(N_i)$)] the sampling variance,

$$\mathrm{Var}(\hat{\mu} + \frac{1}{2}\hat{\sigma}^2) = \mathrm{Var}(\hat{\mu}) + \mathrm{Var}(\frac{1}{2}\hat{\sigma}^2) - 2\mathrm{cov}(\hat{\mu}, \frac{1}{2}\hat{\sigma}^2) \tag{S4}$$

We estimate each component of the variance of the expected number of individuals in order to identify the component that contributes the most to the sampling variance.

The sampling variance of the total expected number of individuals (i.e., $E(N)$, Eq. S4) was estimated to be 2.272. The variance of $\frac{1}{2}\hat{\sigma}^2$ was the highest (1.307) while the variance of μ was lower (0.523) and the covariance between μ and $\frac{1}{2}\hat{\sigma}^2$ was negative (-0.442). It is the variance in species unevenness which contribute the most to variance to the total expected number of individuals.

Estimation of the interannual fluctuations of species diversity

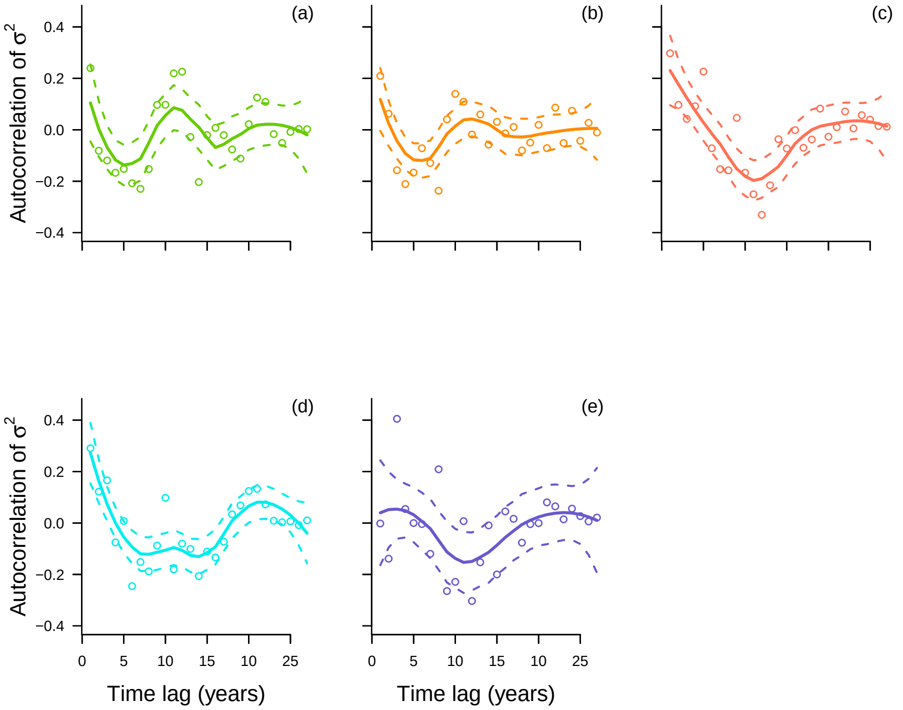

Figure S8: Temporal correlation pattern for unevenness as a function of monthly time lag in (a) June (b) July (c) August (d) September (e) October. The solid line represents the smoothed curve of the unevenness temporal autocorrelation and the dotted line represents the 95 % confidence intervals.

Online Resource 5

Table S2: Estimated values for the variance decomposition of the total variance of the lognormal species abundance distribution and return time to equilibrium (Eq. 1 and Eq. 4).

	June	July	August	September	October
$\hat{\sigma}_{heter}$	0.88	1.27	2.04	3.00	2.65
$\hat{\sigma}_{stoch}$	1.95	2.01	2.76	2.06	1.90
$\hat{\theta}$	0.12	0.14	0.20	0.21	0.19
$\frac{1}{\hat{\delta}}$	0.79	0.53	0.69	0.30	0.37

Table S3: Linear mixed effect model ($n = 110$) that describes the relationships between half of the species diversity ($\frac{1}{2}\sigma^2$) and the expected number of individuals ($\ln(EN)$) including the sampling period as random effect (Fig. 6).

	Estimate	Std.Error	t value	Pr(> | t |)
Intercept	6.215	0.264	23.471	0
$\frac{1}{2}\sigma^2$	0.743	0.634	11.726	0

Generalized additive mixed effect model

Generalized additive mixed model (GAMM) estimate additive non-parametric functions by smoothing splines to model covariate effects (Wood, 2006). A generalized additive mixed model (GAMM) is a generalized linear mixed model (GLMM) in which part of the linear predictor is specified in terms of smooth function of covariates (Lin and Zhang, 1999) such as,

$$y_i = X_i\beta + f_1(x_{1i}) + f_2(x_{2i}, x_{3i}) + Z_i b + \epsilon_i \quad \text{(S5)}$$

where y_i is a univariate response at the ith observation. The vector of fixed parameters is β; The row of fixed effects model matrix is X_i. The f_j are smooth function of covariates x_k; Z_i is a row of a random vector effects model matrix; $b \sim N(0, \Psi_\theta)$ is a vector of random effects coefficients with unknown positive definite covariance matrix Ψ_θ with parameter θ and ϵ_i is a residual error vector with a normal distribution.

Environmental predictors were initially included as smooth terms using penalized regression splines with maximally 4 knots (i.e., 3 degrees of freedom)(Wood and Augustin, 2002). To obtain our final GAMM model we used an hypothesis testing procedure by dropping the least significant term from the model and refit the model until all terms are significant and the AIC being minimal (Zuur et al., 2009). We estimated the GAMM by using the function `gamm` of the `mgcv` R library.

GAMM of the species diversity and environmental variables

Table S4: Generalized additive mixed models ($n = 140$) relating species diversity (σ^2) and environmental predictors in the Lake Atnsjøen (Norway) between June and October from 1990 to 2017. The $\times$ represents the integration of the variables in the model.

	Model Rank					
Variables	1	2	3	4	5	6
Temperature	×	×	×	×	×	×
Water transparency	×	×	×	×	×	
Year	×	×	×	×		
Ice Cover	×	×	×			
Phytoplankton	×	×				
River run-off	×					

Table S5: The respective Akaike's Information Criterion (AIC) values and Δ AIC are presented for each model presented in Table S4.

Model rank	AIC	Δ AIC
1	620.032	10.475
2	616.040	6.483
3	612.459	2.902
4	609.557	0
5	610.705	1.148
6	614.241	4.684

Table S6: Generalized additive mixed models ($n = 112$) relating species diversity (σ^2) and environmental predictors as well as 1-month lagged environmental predictors in the Lake Atnsjøen (Norway) between July and October from 1990 to 2017. The $\times$ represents the integration of the variables in the model.

	Model Rank							
Variables	1	2	3	4	5	6	7	8
Temperature	×	×						
Phytoplankton	×	×	×	×	×			
Ice Cover	×	×	×	×	×	×		
River run off	×	×	×					
Water transparency	×	×	×	×	×	×	×	
Temperature $(t-1)$	×	×	×	×	×	×	×	×
Phytoplankton $(t-1)$	×	×	×	×	×	×	×	×
River run-off $(t-1)$	×	×	×	×				
Water transparency $(t-1)$	×							

Table S7: The respective Akaike's Information Criterion (AIC) values and Δ AIC are presented for each model presented in Table S6.

Model rank	AIC	Δ AIC
1	517.27	16.630
2	513.29	12.650
3	509.82	9.180
4	506.49	5.857
5	504.94	4.304
6	502.76	2.121
7	500.64	0
8	501.29	0.658

Selected GAMM models

The selected GAMM model (Table S5) of the unevenness included years and water temperature was as follows,

$$\sigma_i^2 = \beta_0 + \beta_1\text{water temperature} + \beta_2\text{water transparency} + \beta_3\text{Year}+ \\ f(\text{water temperature, k=4}) + f(\text{water transparency, k=4}) + f(\text{Year, k=4}) + b + \epsilon_i. \quad \text{(S6)}$$

The selected GAMM model (Table S7) that describes relation between unevenness and 1-month lagged water temperature, 1-month lagged phytoplankton and water transparency was as follows,

$$\sigma_i^2 = \beta_0 + \beta_1\text{1-month lagged water temperature}+ \\ \beta_2\text{1-month lagged phytoplankton}+ \\ + \beta_3\text{water transparency}+ \\ f(\text{1-month lagged water temperature, k=4}) + f(\text{1-month lagged phytoplankton, k=4}) \\ + f(\text{water transparency, k=4}) + \epsilon_i. \quad \text{(S7)}$$

where σ^2 is the unevenness at the ith observation, βs are the coefficient of the fixed effect, f and are smooth functions of the predictor variables. The random effects b (i.e., sampling period effect) and residuals ϵ_i are assumed to be independent and normally distributed. We included additional autocorrelation between observations by introducing an autoregressive correlation structure of order 1 (AR-1) model on the residuals ϵ.

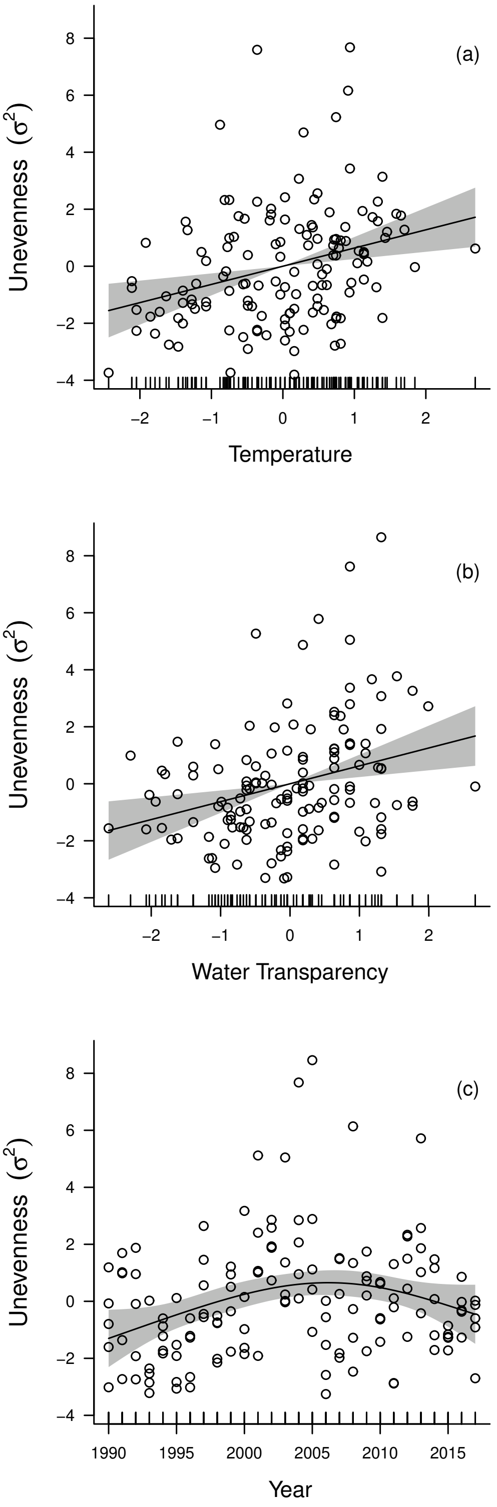

Figure S9: Partial plots of smooth function from a general additive mixed model of the predicted relationships between unevenness (σ^2) and significant environmental variables ($n = 140$, $R^2 = 0.23$, Table S5) (a) temperature (b) water temperature (c) year. Etchings (rugs) on the inside of the x-axis indicate locations of data points along this axis. Grey shaded area represents 95% GAM specific metric credibility intervals.

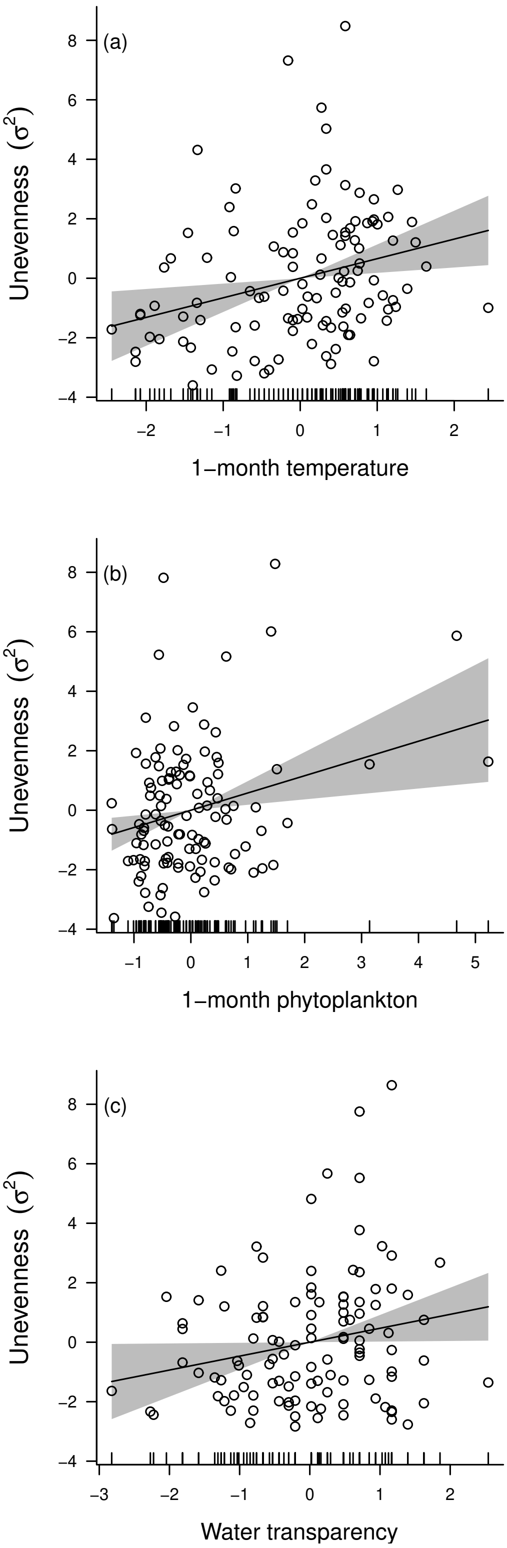

Figure S10: Partial plots of smooth function from a general additive mixed effect model of the predicted relationships between unevenness (σ^2) and 1− month lagged and without lag environmental variables ($n = 112$, $R^2 = 0.17$, Table S7). Etchings (rugs) on the inside of the x axis indicate locations of data points along this axis. Grey shaded area represents 95% GAM specific metric credibility intervals.

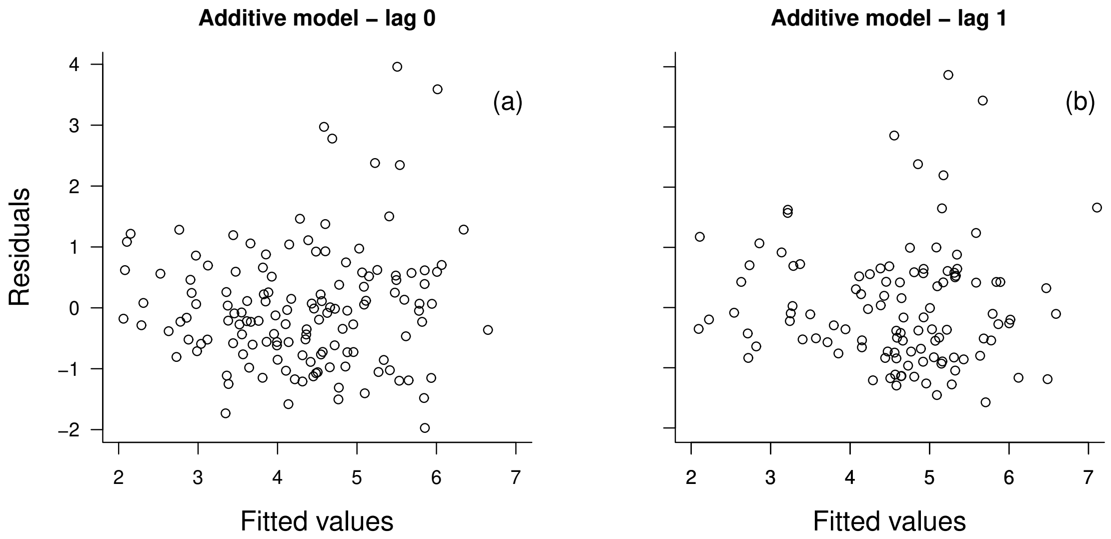

Figure S11: (a) residuals of the GAMM with environmental variables (see Fig. S9);(b) residuals of the GAMM with 1-month lagged environmental variables (see Fig. S10).

Linear trend in water temperature

Table S8: Linear model ($n = 26$, $df = 26$, $R^2 = 0.1801$) that describes the relationships between the water temperature and years in Lake Atnsjøen in July from 1990 to 2017.

	Estimates	Std.Error	t value	Pr(> | t |)
Intercept	-62.1749	29.2531	-2.13	0.0432
Year	0.0349	0.0146	2.39	0.0244

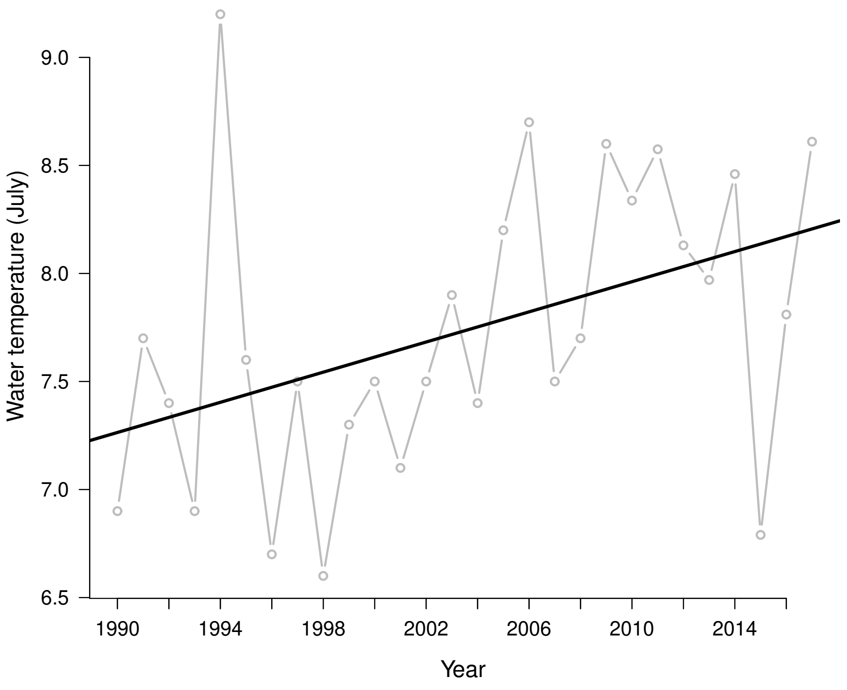

Figure S12: Relationships between water temperature and years. Dotted grey line: measured water temperature in July from 1990 to 2017; solid line: linear model between temperature and years (Table S8).

Interannual variation in standardized abundance of the most common species

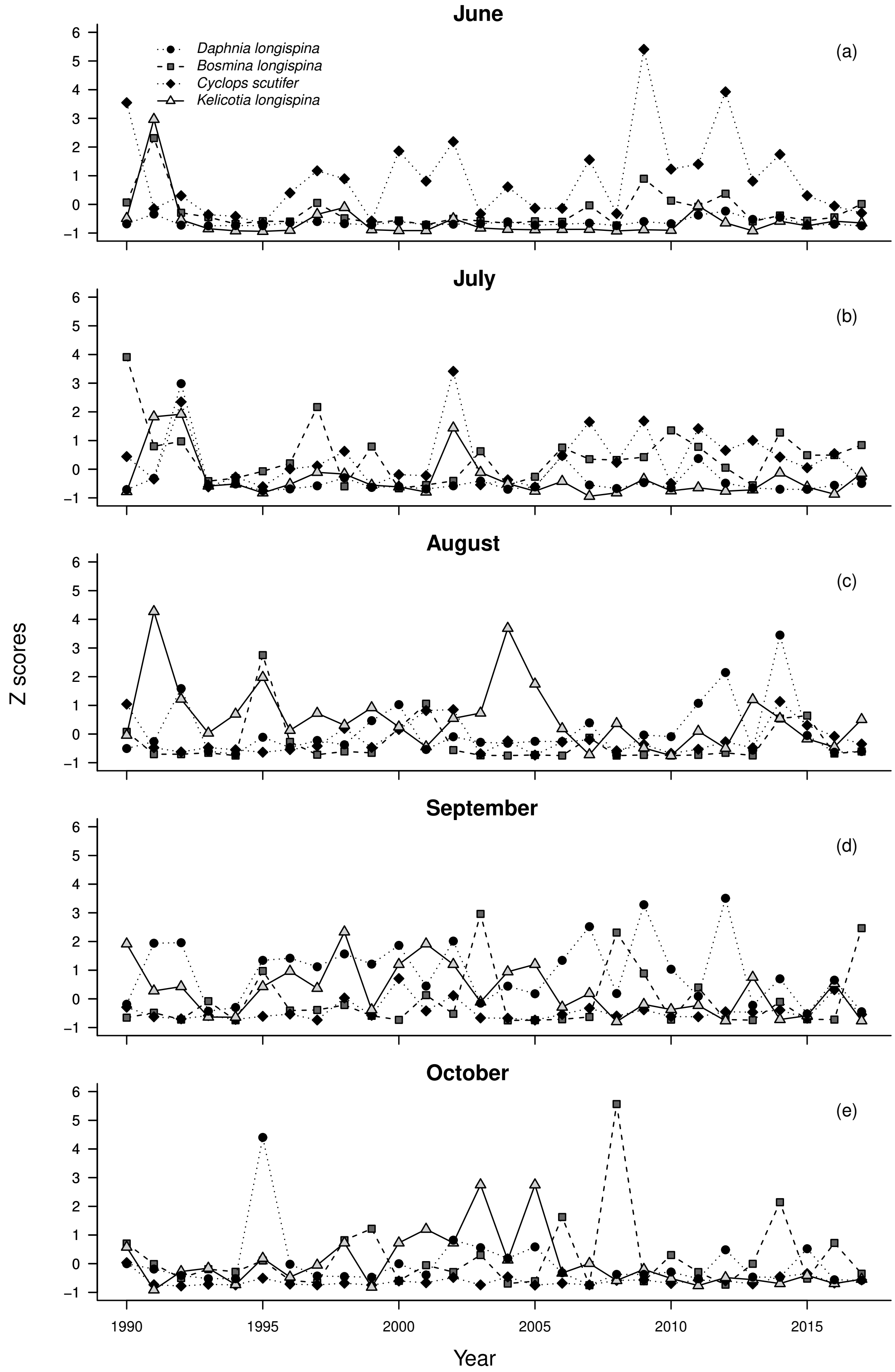

Figure S13: Zscores of the most common species through time for each sampling period from 1990 to 2017. A zscore gives an estimation of how much a given data point (here the standardized abundances of a given species) is far from the mean (i.e., mean abundances of a given species over the duration of the study).

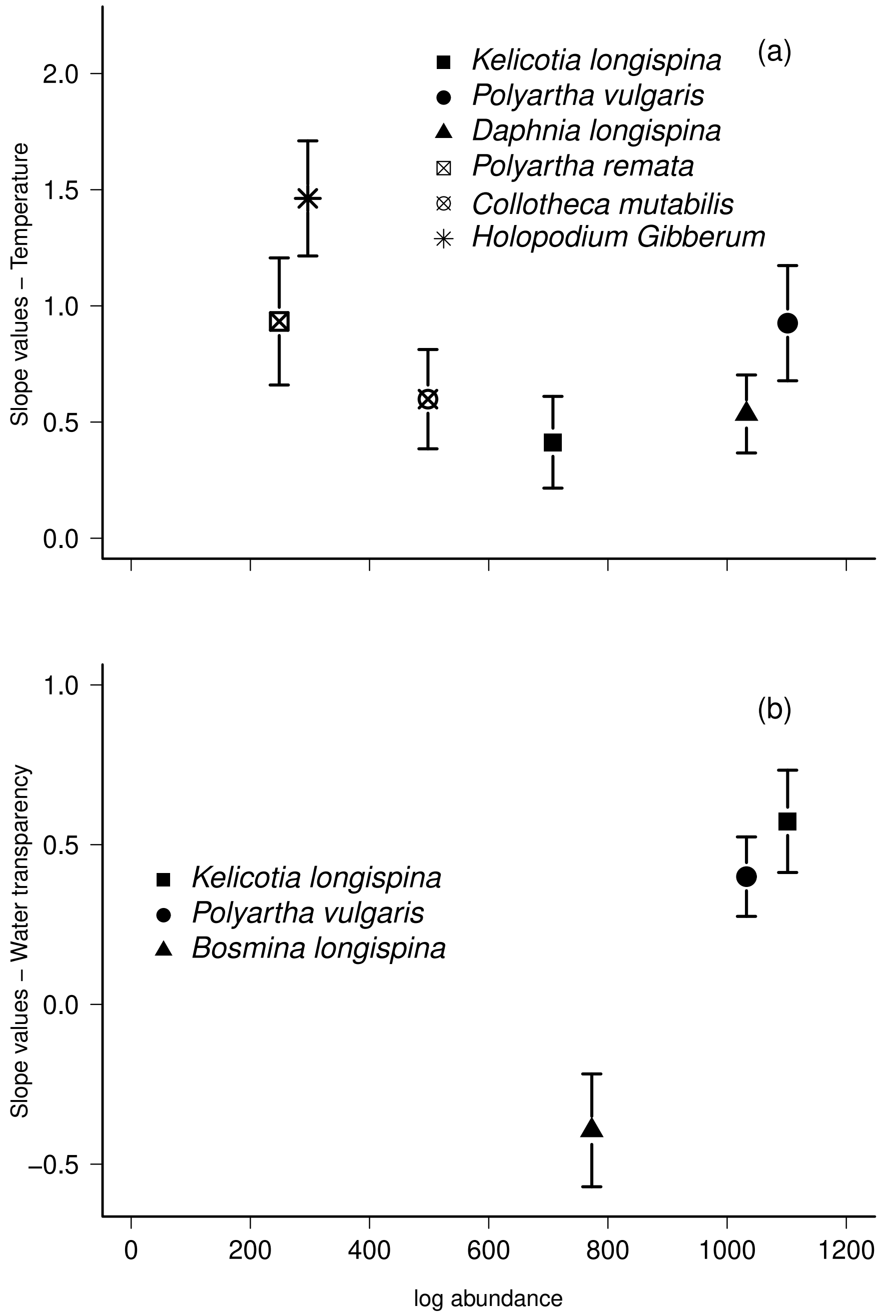

Figure S14: Estimated values of the slope and their 95% confidence intervals of the species for which their fluctuations in abundance over time are significantly explained by (a) the temperature and (b) the water transparency. The estimated slopes were issued from generalized linear models which expressed log abundance of species as function of temperature and water transparency.

References

Brettum P, Halvorsen G (2004) The phytoplankton of lake atnsjoen, norway - a long-term investigation. Hydrobiologia 521(1-3):141–147

Bulmer M (1974) On fitting the poisson lognormal distribution to species abundance data. Biometrics 30:651–660

Engen S, Lande R (1996) Population dynamic models generating the lognormal species abundance distribution. Mathematical Biosciences 173:85–102

Engen S, Lande R, Walla T, DeVries PJ (2002) Analyzing spatial structure of communities using the two-dimensional poisson lognormal species abundance model. The American Naturalist 160(1):60–73

Engen S, Aagaard K, Bongard T (2011) Disentangling the effects of heterogeneity, stochastic dynamics and sampling in a community of aquatic insects. Ecological Modelling 222(8):1387–1393

Fisher R, Corbet A, Williams C (1943) The relation between the number of species and the number of individuals in a random sample from an animal population. Journal of Animal Ecology 12:42–58

Gillooly JF (2000) Effect of body size and temperature on generation time in zooplankton. Journal of Plankton research 22(2):241–251

Grøtan V, Engen S (2008) poilog: Poisson lognormal and bivariate Poisson lognormal distribution. R package version 0.4

Hessen DO, Faafeng BA, Smith VH, Bakkestuen V, Walseng B (2006) Extrinsic and intrinsic controls of zooplankton diversity in lakes. Ecology 87(2):433–443

Karlin S, Taylor H (1981) A second course in stochastic processes. Academic Press, New York

Lande R, Engen S, Sæther BE (2003) Stochastic Population Dynamics in Ecology and Conservation. Oxford Series in Ecology and Evolution, Oxford University Presss, Oxford

Lin X, Zhang D (1999) Inference in generalized additive mixed models using smoothing splines. Journal of the Royal Statistical Society, Series B 61:381–400

Mazumder A, Taylor WD, McQueen DJ, Lean DRS (1990) Effects of fish and plankton on lake temperature and mixing depth. Science 247(4940):312–315

Tvede AM (2004) Hydrology of lake atnsjoen and river atna. Hydrobiologia 521(1-3):21–34

Walsh SE, Vavrus SJ, Foley JA, Fisher VA, Wynne RH, Lenters JD (1998) Global patterns of lake ice phenology and climate: Model simulations and observations. Journal of Geophysical Research-Atmospheres 103(D22):28,825–28,837

Williams CB (1964) Patterns in the balance of nature and related problems in quantitative ecology. Academic Press, London

Wood SN (2006) Generalized Additive Models. An introduction with R. Chapman et Hall

Wood SN, Augustin NH (2002) Gams with integrated model selection using penalized regression splines and applications to environmental modelling. ecological modelling 157(2-3):157–177

Zuur A, Ieno EN, Walker N, Saveiliev AA, Smith GM (2009) Mixed Effects Models and Extensions in Ecology with R. Springer
